# Characterizing tumor biology and immune microenvironment in high-grade serous ovarian cancer via single-cell RNA sequencing: insights for targeted and personalized immunotherapy strategies

**DOI:** 10.3389/fimmu.2024.1500153

**Published:** 2025-01-17

**Authors:** Fu Zhao, Xiaojing Jiang, Yumeng Li, Tianjiao Huang, Zhikai Xiahou, Wenyang Nie, Qian Li

**Affiliations:** ^1^ Xiyuan Hospital of China Academy of Chinese Medical Sciences, Beijing, China; ^2^ Shandong University of Traditional Chinese Medicine, Jinan, China; ^3^ Affiliated Hospital of Shandong Academy of Traditional Chinese Medicine, Jinan, China; ^4^ The First School of Clinical Medicine, Heilongjiang University of Traditional Chinese Medicine, Harbin, China; ^5^ China Institute of Sport and Health Science, Beijing Sport University, Beijing, China

**Keywords:** high-grade serous ovarian cancer (HGSOC), prognostic model, immunotherapy, molecular mechanisms, tumor microenvironment, multi-omics

## Abstract

**Background:**

High-grade serous ovarian cancer (HGSOC), the predominant subtype of epithelial ovarian cancer, is frequently diagnosed at an advanced stage due to its nonspecific early symptoms. Despite standard treatments, including cytoreductive surgery and platinum-based chemotherapy, significant improvements in survival have been limited. Understanding the molecular mechanisms, immune landscape, and drug sensitivity of HGSOC is crucial for developing more effective and personalized therapies. This study integrates insights from cancer immunology, molecular profiling, and drug sensitivity analysis to identify novel therapeutic targets and improve treatment outcomes. Utilizing single-cell RNA sequencing (scRNA-seq), the study systematically examines tumor heterogeneity and immune microenvironment, focusing on biomarkers influencing drug response and immune activity, aiming to enhance patient outcomes and quality of life.

**Methods:**

scRNA-seq data was obtained from the GEO database in this study. Differential gene expression was analyzed using gene ontology and gene set enrichment methods. InferCNV identified malignant epithelial cells, while Monocle, Cytotrace, and Slingshot software inferred subtype differentiation trajectories. The CellChat software package predicted cellular communication between malignant cell subtypes and other cells, while pySCENIC analysis was utilized to identify transcription factor regulatory networks within malignant cell subtypes. Finally, the analysis results were validated through functional experiments, and a prognostic model was developed to assess prognosis, immune infiltration, and drug sensitivity across various risk groups.

**Results:**

This study investigated the cellular heterogeneity of HGSOC using scRNA-seq, focusing on tumor cell subtypes and their interactions within the tumor microenvironment. We confirmed the key role of the C2 IGF2+ tumor cell subtype in HGSOC, which was significantly associated with poor prognosis and high levels of chromosomal copy number variations. This subtype was located at the terminal differentiation of the tumor, displaying a higher degree of malignancy and close association with stage IIIC tissue types. The C2 subtype was also associated with various metabolic pathways, such as glycolysis and riboflavin metabolism, as well as programmed cell death processes. The study highlighted the complex interactions between the C2 subtype and fibroblasts through the MK signaling pathway, which may be closely related to tumor-associated fibroblasts and tumor progression. Elevated expression of PRRX1 was significantly connected to the C2 subtype and may impact disease progression by modulating gene transcription. A prognostic model based on the C2 subtype demonstrated its association with adverse prognosis outcomes, emphasizing the importance of immune infiltration and drug sensitivity analysis in clinical intervention strategies.

**Conclusion:**

This study integrates molecular oncology, immunotherapy, and drug sensitivity analysis to reveal the mechanisms driving HGSOC progression and treatment resistance. The C2 IGF2+ tumor subtype, linked to poor prognosis, offers a promising target for future therapies. Emphasizing immune infiltration and drug sensitivity, the research highlights personalized strategies to improve survival and quality of life for HGSOC patients.

## Introduction

High-grade serous ovarian cancer (HGSOC) is the most common subtype of epithelial ovarian cancer (OCa), responsible for about 185,000 deaths globally each year, representing approximately 60% of all OCa-related fatalities (1, 2). The ovaries’ unique anatomical location leads to subtle early symptoms of HGSOC, causing around 70% of patients to be diagnosed at advanced stages (III/IV) with pelvic-abdominal dissemination (3). Research shows that patients with aggressive stage IIIC tumors have a 5-year survival rate of 15% to 25%, which significantly affects their quality of life (4).

Currently, the standard treatment regimen for HGSOC typically involves the combination of primary debulking surgery and platinum-based chemotherapy (5). primary debulking surgery aims to maximize tumor burden reduction to enhance the efficacy of subsequent chemotherapy, while platinum-based chemotherapy remains central to the treatment of HGSOC (1, 6). However, despite showing a certain degree of positive response to platinum-based chemotherapy in initial treatment for advanced HGSOC (7), there has been no significant improvement in cure rates over the past 30 years. This stagnation is primarily attributed to the anatomical continuity of the abdominal cavity, which facilitates early dissemination of cancer cells (8). Furthermore, although novel therapeutic agents such as bevacizumab and PARP inhibitors have been introduced in recent years, survival outcomes for patients with stage IIIC or higher who receive neoadjuvant chemotherapy (9–11). These challenges highlight the need to explore molecular mechanisms driving treatment resistance and to develop personalized therapeutic strategies that incorporate drug sensitivity analysis and immunotherapy. By integrating these insights, future treatments may significantly enhance patient outcomes in HGSOC, improving both survival and quality of life.

The rapid advancement of immunotherapy has revolutionized tumor-targeted cancer treatment. The diverse organization and maturity of immune aggregates identified in HGSOC provide critical insights for the development of alternative immunotherapeutic strategies tailored for HGSOC patients (12). Moreover, in the treatment strategies for recurrent HGSOC, the combination of pathway blockade and checkpoint inhibition has demonstrated significant therapeutic potential (13). Despite these advancements, some studies suggest that HGSOC has a suboptimal response to immunotherapy. This may be due to its tumor mutational burden, which is significantly lower than that seen in other tumor types, ultimately reducing the disease’s inherent immunogenicity. Consequently, the field of immunotherapy for HGSOC remains contentious, with existing therapeutic outcomes lacking robust clinical validation. Given the complexity of HGSOC and the limitations of current therapeutic outcomes, there is an urgent need to explore novel immunotherapeutic approaches and multidisciplinary collaborative strategies to improve patient prognosis and quality of life.

Recent advances in single-cell RNA sequencing (scRNA-seq) technology have created important new tools for studying tumor cell heterogeneity and associated microenvironments. This technique enables researchers to characterize gene expression patterns at high resolution, even when analyzing a limited number of cells. Based on this background, we conducted a scRNA-seq study on HGSOC cells, aiming to elucidate the cellular heterogeneity and microenvironmental characteristics of HGSOC, thereby providing novel insights for its diagnosis and treatment. By revealing the transcriptomic profiles of various cell types, we anticipate identifying potential biomarkers and therapeutic targets that could significantly enhance patient prognosis and survival rates. This study aims to establish a foundation for developing personalized treatment strategies to manage the complexities and challenges of HGSOC.

## Materials and methods

### Get HGSOC data

The scRNA-seq data for HGSOC was obtained from the GEO database (https://www.ncbi.nlm.nih.gov/geo/). The dataset used in the single-cell analysis included ovarian samples from five normal ovarian disease patients with six HGSOC patients, with the accession number GSE184880 (GSM5599220-GSM5599231). Detailed clinical data from these patients with metastatic omental tumors, such as age, histologic type, tumor stage, BRCA/HRR status (nucleotide change) were provided. For more information, refer to **Supplementary Table 1**. Additionally, bulk RNA-seq data and clinical data were obtained from the Cancer Genome Atlas (TCGA) (https://portal.gdc.cancer.gov/), which also included genetic mutation data and clinical information, such as patient survival details for ovarian cancer.

### Processing and visualization of raw data

The analysis of 10X genomics data for each sample was conducted using R software (version 4.2.0) in conjunction with the Seurat package (v4.1.1) (14, 122). To maintain data integrity, we utilized the DoubletFinder tool (v2.0.3) to detect and eliminate probable doublet cells (15–17). Furthermore, inferior-quality cells were excluded to improve the precision and dependability of the scRNA-seq results. Cells were incorporated into the study if they satisfied the subsequent criteria: 300 < nFeature < 6000 and 500 < nCount < 100,000. Moreover, the expression of mitochondrial genes in each cell constituted less than 25% of the overall gene expression, whereas the expression of red blood cell genes was confined to under 5% of the total.

Following filtration, samples were normalized with the “NormalizeData” tool, and the top 2,000 highly variable genes were identified using the “FindVariableFeatures” function from the Seurat package (18–22). The produced data were further standardized using the “ScaleData” function, followed by principal component analysis (PCA) (23–25). We mitigated batch effects across datasets by employing the harmony R package (version 0.1.1) (26, 27). We subsequently identified the top 30 principal components for additional analysis and displayed the data with uniform manifold approximation and projection (UMAP) (28, 121).

### Cancer preferences analysis

To evaluate the cancer preference of tumor cell subtypes, odds ratios (OR) were computed utilizing the specified calculation method (29).

### Enrichment analysis and AUCell analysis

We performed a functional analysis of Gene Ontology Biological Process (GOBP) (30–33) and Kyoto Encyclopedia of Genes and Genomes (KEGG) utilizing the ClusterProfiler R package (version 4.6.2) based on Gene Ontology (GO) analysis (34, 35). Gene Set Enrichment Analysis (GSEA) was utilized to assess the comprehensive expression patterns within gene sets (36). Additionally, we integrated a novel method called AUCell to detect active gene sets within our scRNA-seq data (37). AUCell is a computational method used to assess gene set enrichment in single-cell transcriptomic data. We evaluated the enrichment of stemness gene sets using the AUCell R package, ranking the gene sets according to the degree of enrichment with the “AUCell_buildRankings” function.

A method used for gene set enrichment analysis is gene set variation analysis (GSVA). This technique assessed the variability of gene expression data and compares it to predefined gene sets to calculate enrichment scores for each gene set in every sample.

### Detect tumor cells utilizing inferCNV

We conducted CNV analysis of the scRNA-seq data using the R package infercnv (version 1.6.0, https://github.com/broadinstitute/inferCNV). This analysis involved evaluating relative gene expression alongside chromosomal location data to infer the CNV status of chromosomes in individual cells (38). This approach enabled us to distinguish malignant tumor cells from normal cells effectively.

### Cell type identification and annotation

We utilized Seurat’s “FindClusters” and “FindNeighbors” functions for cell clustering, and “FindAllMarkers” to identify Differentially Expressed Genes (DEGs) for each cluster (39–41). Subsequently, to further investigate the heterogeneity of tumor cells in HGSOC, we re-clustered the tumor cells and marked each subtype based on specific marker genes.

### Lineage trajectory analysis

To assess the differentiation states of all tumor cell subtypes, we utilized CytoTRACE for ranking (42). We constructed the pseudotime trajectory of these subtypes using Monocle (v2.24.0). By analyzing the pseudotime ordering within the scRNA-seq data, we then leveraged the Slingshot package (version 2.6.0) to infer cell lineages during the differentiation process of the tumor cell subtypes (43). Using the “getLineages” and “getCurves” functions, we estimated the expression levels associated with each lineage, thereby elucidating the differentiation trajectories of the tumor cell subtypes.

### Cell communication

We employed the CellChat R package (version 1.6.1) (34) to visualize interactions among all cell types, including communication networks between tumor subtypes and other cell types. This involved quantitatively inferring and analyzing intercellular interactions based on scRNA-seq data. We used the “netVisual_diffInteraction” function to depict differences in cell communication intensity and the “identifyCommunicationPatterns” function to identify various communication patterns. We utilized the CellChat database to predict signaling pathways and ligand-receptor interactions. A p-value threshold of 0.05 was set to evaluate intercellular interactions among various cell types.

### Utilization of SCENIC for gene regulatory network reconstruction

We utilized the pySCENIC package (v0.10.0) in Python (v3.7) with default parameters to reconstruct gene regulatory networks and identify stable cell states from scRNA-seq data. We generated AUCell matrices to assess transcription factor enrichment and regulatory factor activity (44, 45).

### Construction and validation of prognostic model

Initially, we found notable predictive genes via univariate Cox analysis and Lasso regression analysis (46). We subsequently computed the risk coefficients for each prognostic gene by multivariate Cox regression analysis and developed a risk score model (Risk score =∑_i^n Xi×Yi, where X represents the coefficient and Y denotes the gene expression level) (47). Subsequently, we categorized patients according to the optimal cut-off value established by the median risk score and the “surv_cutpoint” function. We performed survival analysis utilizing the Survival package in R (version 3.3.1-1) to examine the prognostic outcomes of various patient cohorts and depicted the survival curves using the ggsurvplot function (48–51). Subsequently, we assessed the predictive model’s accuracy by generating ROC curves (52–55) utilizing the timeROC package (version 0.4.0).

Additionally, to confirm the independence of the risk score as a prognostic indicator, we conducted multivariate Cox regression analysis and developed a Nomogram to forecast overall survival (OS) at 1, 3, and 5 years. We performed internal cross-validation of the Nomogram’s predictions utilizing the C-index and calibration curves.

### Assessment of immune microenvironment

We utilized the CIBERSORT R software (version 0.1.0) to evaluate 22 categories of immune cells. Subsequently, we employed CIBERSORT, ESTIMATE, and Xcell methods to thoroughly assess the immunological microenvironment of the patients, and further examined the variations in immune cell infiltration levels and the expression levels of immune checkpoint-related genes (56).

Furthermore, we examined the relationships among immune cells, model genes, OS, and risk ratings. We evaluated the response to tumor immunotherapy utilizing the Tumor Immune Dysfunction and Exclusion (TIDE) program (http://tide.dfci.harvard.edu). We assessed medication immune response utilizing the TCIA database (https://www.cancerimagingarchive.net/).

### Drug sensitivity analysis

To better align our findings with the clinical application of the drugs, we assessed the sensitivity of various drugs. We employed the “pRRophetic” package (version 0.5) to determine the IC50 values for each sample and to evaluate the responsiveness of groups with high and low risk scores (57, 58, 123).

### Cell culture

The OVCAR3 cell line (Catalog No.: CL-0178), derived from tumor tissue, was purchased from Wuhan PunoSai Life Technology Co., Ltd. It was cultivated in RPMI-1640 medium under controlled environmental conditions, which included a temperature of 37°C, a 5% CO2 atmosphere, and a humidity level of 95%. The medium was also supplemented with 10% fetal bovine serum (FBS) and 1% antibiotics to support cell proliferation and maintain cell health. In a similar fashion, the OVCAR8 cell line was also grown in RPMI-1640 medium under the same environmental parameters, with the addition of 10% FBS and 1% antibiotics to promote optimal cellular growth and vitality.

### Cell transfection

RNA constructs procured from GenePharma (Suzhou, China) were utilized to suppress the expression of PRRX1. The cells were plated in a 6-well plate at a density of 50% confluence and then underwent transfection with PRRX1-targeting RNAi constructs (si-PRRX1-1 and si-PRRX1-2) and a non-targeting control (si-NC). The transfection process was facilitated by Lipofectamine 3000RNAiMAX reagent (Invitrogen, USA) following the provider’s protocol. A range of si-RNAs (RIbbio, China) were introduced into the cells. The sequences of the si-RNAs, presented from the 5’ to 3’ direction, are detailed in **Supplementary Table 2**.

### Western blotting

Once the cells reached approximately 70% confluence post-transfection, they were lysed using RIPA buffer for harvesting. The resulting lysates were subjected to centrifugation at 12,000 rpm for 15 minutes to remove cellular debris, and the supernatants were collected for subsequent protein analysis using SDS-PAGE. The separated proteins were then transferred to PVDF membranes, which were incubated with 5% bovine serum albumin (BSA) for 1.5 hours at room temperature to minimize non-specific interactions. Following this, the membranes were exposed to a primary Anti-PRRX1 antibody overnight at 4°C, and subsequently incubated for one hour with a secondary antibody conjugated to horseradish peroxidase. The detection of PRRX1 protein was conducted by applying an enhanced chemiluminescence substrate for Western Blotting.

### Quantitative real-time polymerase chain reaction

RNA was extracted utilizing Trizol reagent, followed by a reverse transcription process performed with the PrimeScript™ Kit. To quantify gene expression, qRT-PCR analysis was performed using SYBR Green as the fluorescent dye to track the amplification progress.

### Cell viability assay

To assess the impact of transfection on the viability of OVCAR3 and OVCAR8 cells, the CCK-8 assay was utilized. Cells were seeded in 96-well plates at a density of 5×10³ cells per well and incubated for 24 hours. Subsequently, 10μL of CCK-8 reagent (A311-01, Vazyme) was added to each well, and the plates were incubated in the dark at 37°C for two hours to allow the colorimetric reaction to develop. Absorbance readings at 450 nm were taken daily over a four-day period using a spectrophotometric plate reader (A33978, Thermo) to evaluate cell viability. The mean optical density values were calculated and plotted to demonstrate the cell survival trend throughout the observation period.

### Transwell assay

Prior to commencing the experiment, cells underwent serum starvation for a duration of 24 hours in a serum-free medium. Following this, the cells were mixed with Matrigel (BD Biosciences, USA) and introduced into the upper chamber of Costar transwell plates, while the lower chamber was filled with a medium supplemented with serum to create a chemotactic gradient. The plates were then incubated for 48 hours to promote migration and invasion of the cells. After the incubation, the cells were fixed using 4% paraformaldehyde and subsequently stained with crystal violet to assess their invasive potential.

### Wound-healing assay

For the assessment of cell migration in stably transfected cells, they were cultured in 6-well plates until confluence was achieved. A sterile 200μL pipette tip was used to create even scratches in the cell monolayer within each well. The wells were then rinsed with PBS to clear away any detached cells or debris. In light of this, the cells were incubated in serum-free medium to observe their migratory response. Photographs of the wounded areas were taken at the 0-hour mark and again after a 48-hour incubation period. The Image-J software was employed to measure the scratch widths and perform a quantitative analysis of the cell migration.

### 5-ethynyl-2’-deoxyuridine proliferation assay

Following transfection, OVCAR3 and OVCAR8 cells were seeded in 6-well plates at a density of 5×10³ cells per well. After a 24-hour incubation at room temperature, EdU reagent was introduced into the culture medium, and the cells were incubated for an additional 2 hours to facilitate DNA labeling during the S phase. Subsequently, the cells were washed twice with PBS to eliminate any unincorporated EdU. They were then fixed in a 4% paraformaldehyde solution for 15 minutes. After fixation, the cells underwent permeabilization, followed by treatment with a glycine/Triton X-100 solution for 15 minutes to prevent non-specific staining. Finally, the cells were stained with a combination of 1X Apollo and Hoechst dyes for 30 minutes. Fluorescence microscopy was employed to visualize and capture images of the stained cells, enabling the assessment of cell proliferation.

### Statistical analysis

Statistical analyses were performed utilizing R software (v4.3.0) and Python software (v4.2.0) (59, 124). We employed Wilcoxon’s test and the Pearson correlation coefficient to evaluate the significance of differences between various groups. Significance levels were classified as follows: *P < 0.05, **P < 0.01, ***P < 0.001, and ****P < 0.0001. The designation “ns” was used to indicate non-significant differences between groups. These statistical tests and significance indicators were applied to assess the statistical validity of our findings and to enhance confidence in the results.

## Results

### Single cell landscape of HGSOC

**Figure 1:**
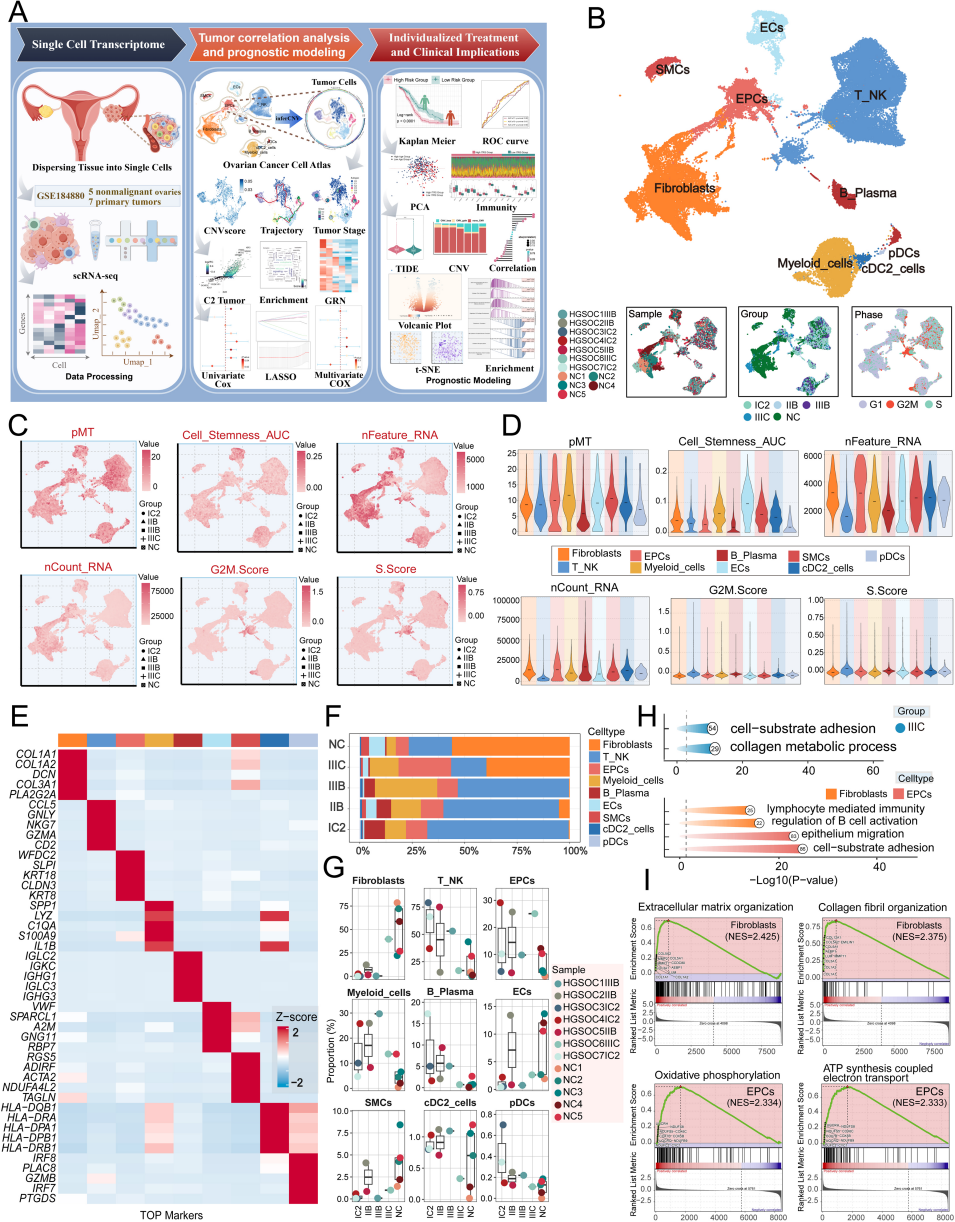
Single-cell profiling of HGSOC. **(A)** Workflow diagram for single-cell sequencing analysis of the GSE184880 dataset. **(B)** The UMAP plot depicted the distribution of nine distinct cell types across the entire cell population, including Fibroblasts, T_NK cells, EPCs, Myeloid cells, B Plasma cells, ECs, SMCs, cDC2, and pDCs. The UMAP plots below illustrated the distribution of twelve distinct samples (left), various tissue types (middle), and different cell cycle phases (right) within the entire cell population. **(C)** The UMAP plots displayed the distribution in pMT, Cell Stemness AUC, nFeature RNA, nCount RNA, G2M.Score, and S.Score. Different group types were distinguished in the figure using various symbols. **(D)** The violin plots illustrated the expression levels of different cell types in terms of pMT, Cell Stemness AUC, nFeature RNA, nCount RNA, G2M.Score, and S.Score. **(E)** The heatmap showed the expression of top five marker gene in different cell types. **(F)** The stacked bar graph illustrated the proportion of each cell type across different groups. **(G)** The box plots illustrated the distribution of each sample across the nine cell types. **(H)** Visualization of enrichment analysis in IIIC group, fibroblasts and EPCs. **(I)** The GSEA enrichment analysis demonstrated the pathways that were upregulated in fibroblasts and EPCs.

We conducted an extensive analysis of the obtained dataset to unveil the intricate single-cell landscape within the HGSOC microenvironment. Our workflow was illustrated in **Figure 1A**. By analyzed five nonmalignant ovaries and seven primary tumors from GSE 184880, By using dimensionality reduction clustering with UMAP plot, nine cell types were obtained: fibroblasts, T-NK cells, epithelial cells (EPCs), myeloid-cells, B-plasma cells, endothelial cells (ECs), smooth muscle cells (SMCs), conventional dendritic cells type 2 (cDC2-cells), plasmacytoid dendritic cells (pDCs).

The figure presented below demonstrated the distribution of twelve distinct samples, as well as the distribution of cells across the five groups (IC2, IIB, IIIB, IIIC, NC) and the three different phases (G1, G2/M, S) in various cell types (**Figure 1B**).

Afterward, we conducted an analysis on the distribution and densities of five cell groups expressing pMT, Cell Stemness AUC, nFeature RNA, nCount RNA, G2/M.Score, and S.Score (**Figure 1C**). Additionally, we visually depicted the expression levels of different cell types in these terms through **Figure 1D**. Furthermore, we provided a detailed description of the expression level of typical marker genes associated with cell subtypes in the cells, as shown in **Figure 1E**. After analyzing the proportions of each cell type across different groups and the distributions of each sample across the nine cell types, we found that fibroblasts and EPCs were the main components of IIIC (**Figures 1F, G**). To further elucidate the IIIC tissue type and the roles of the more prevalent ECs and fibroblasts within this tissue type in HGSOC. we conducted a functional enrichment analysis. Our findings indicated that the IIIC tissue type was significantly enriched in cell-substrate adhesion and collagen metabolic processes. Furthermore, we observed that fibroblasts exhibited enrichment in lymphocyte-mediated immunity, regulation of B cell activation, EPCs exhibited enrichment in epithelium migration, and cell-substrate adhesion. Additionally, fibroblasts demonstrated upregulation in pathways associated with extracellular matrix organization and collagen fibril organization. In contrast, EPCs were notably enriched in epithelium migration and cell-substrate adhesion, and they showed significant upregulation in the oxidative phosphorylation and ATP synthesis coupled electron transport pathways (**Figures 1H, I**). It is noteworthy that HGSOC primarily originates from the malignant transformation of ovarian EPCs (60). Additionally, oxidative phosphorylation, a metabolic process crucial for energy production, has been found to have a close association with the progression of HGSOC. This indicates that our findings are consistent with the known biological functions associated with HGSOC (61).

### Visualization of tumor cell subtypes in HGSOC

**Figure 2:**
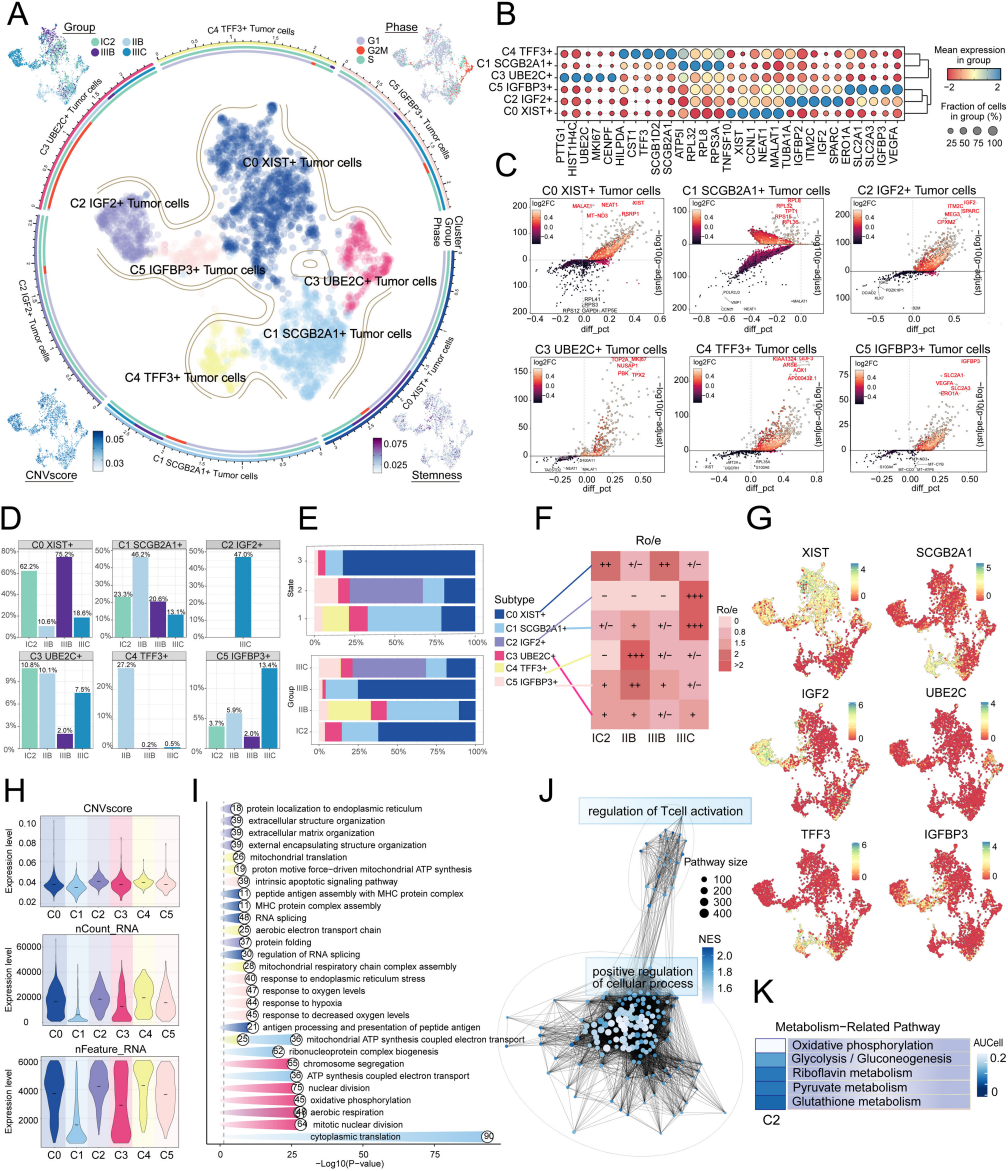
IGF2+ Tumor cells specifically expressed in malignant EPCs and are associated with IIIC. **(A)** The circular plot represented the clustering of six tumor cell subtypes identified in HGSOC, with contour curves delineating the boundaries of each cell subtype. The outer axis displayed a logarithmic scale of the total number of cells within each category. UMAP plots, arranged in the four corners and proceeding clockwise from the upper left corner, illustrated the expression distribution of groups and phases, stemness, and CNV scores across all tumor cells. **(B)** Bubble plots depicted the mean expression levels of the top five DEGs in each tumor cell subtype. The size of each bubble corresponded to the percentage of gene expression, while the color indicated data normalization. **(C)** Volcano plots illustrated significant upregulated and downregulated genes across the six tumor cell subtypes. **(D)** Distribution of groups across different subtypes. **(E)** The stacked bar graphs displayed the distribution of each cell subtype across various states and group classifications. **(F)** The Ro/e score was utilized to assess the tissue preference of each tumor cell subtype. **(G)** UMAP plots revealed the signature marker genes for the six tumor cell subtypes. **(H)** Violin plots depicted the CNV scores, nCount RNA, and Feature RNA expression levels across the six tumor cell subtypes. **(I, J)** Results from enrichment analyses across different group classifications and C2 IGF2+ tumor cells revealed key biological functions of the corresponding cell populations. **(K)** The AUCell algorithm was employed to evaluate the C2 IGF2+ tumor cells in relation to metabolism-related pathways.

Given the profound importance of tumor cells in TME, our subsequent objective is to characterize these cells in the microenvironment of HGSOC. To detect aberrant amplification or deletion of chromosome copy number in EPCs, we initially employed inferCNV to analyze the chromosome copy number variation (CNV) of EPCs using ECs as a reference (**Supplementary Figure 1**). According to CNV level, tumor cells was distinguished from EPCs. After that, we re-subtypeed tumor cells, and annotated them according to each cell marker gene, and identified six tumor cell subtypes: C0 XIST+ tumor cells, C1 SCGB2A1+ tumor cells, C2 IGF2+ tumor cells, C3 UBE2C+ tumor cells, C4 TFF3+ tumor cells and C5 IGFBP3+ tumor cells. We showed the distribution of different groups, phases, stemness, and CNVscore. (**Figure 2A**). **Figure 2B** showcased the differential expression of the top five marker genes in the tumor cell subtypes, visualized using the volcano plots (**Figure 2C**). In addition, we analyzed the proportions between different tissue types and subtypes, finding that the C2 subtype was composed of IIIC. Furthermore, compared to other subtypes, the C2 subtype exhibited a higher proportion of IIIC tissue (**Figures 2D, E**). Therefore, we hypothesized that the heterogeneity about IIIC tissue types may be associated with the C2 subtypes. Similarly, the Ro/e preference plot indicated a higher cell abundance of C2 subtypes in IIIC tissue, further substantiating our conclusion (**Figure 2F**). We also utilized UMAP plots to display the characteristic marker genes of six tumor cell subtypes (**Figure 2G**).

Next, we showed the results of CNVscore, nFeature RNA and nCount RNA of different tissue types and tumor cell subtypes by violin diagrams (**Figure 2H**). Our findings indicated that the C2 subtype exhibited a significantly higher CNV score compared to other subtypes, which is consistent with the biological characteristics associated with HGSOC tissue. Additionally, the C2 subtype demonstrated elevated expression levels of both nCount RNA and nFeature RNA. Consequently, we infer that the C2 subtype may represent a higher level of malignancy.

### Analysis of metabolic and biological processes in tumor cell subtypes

**Figure 3:**
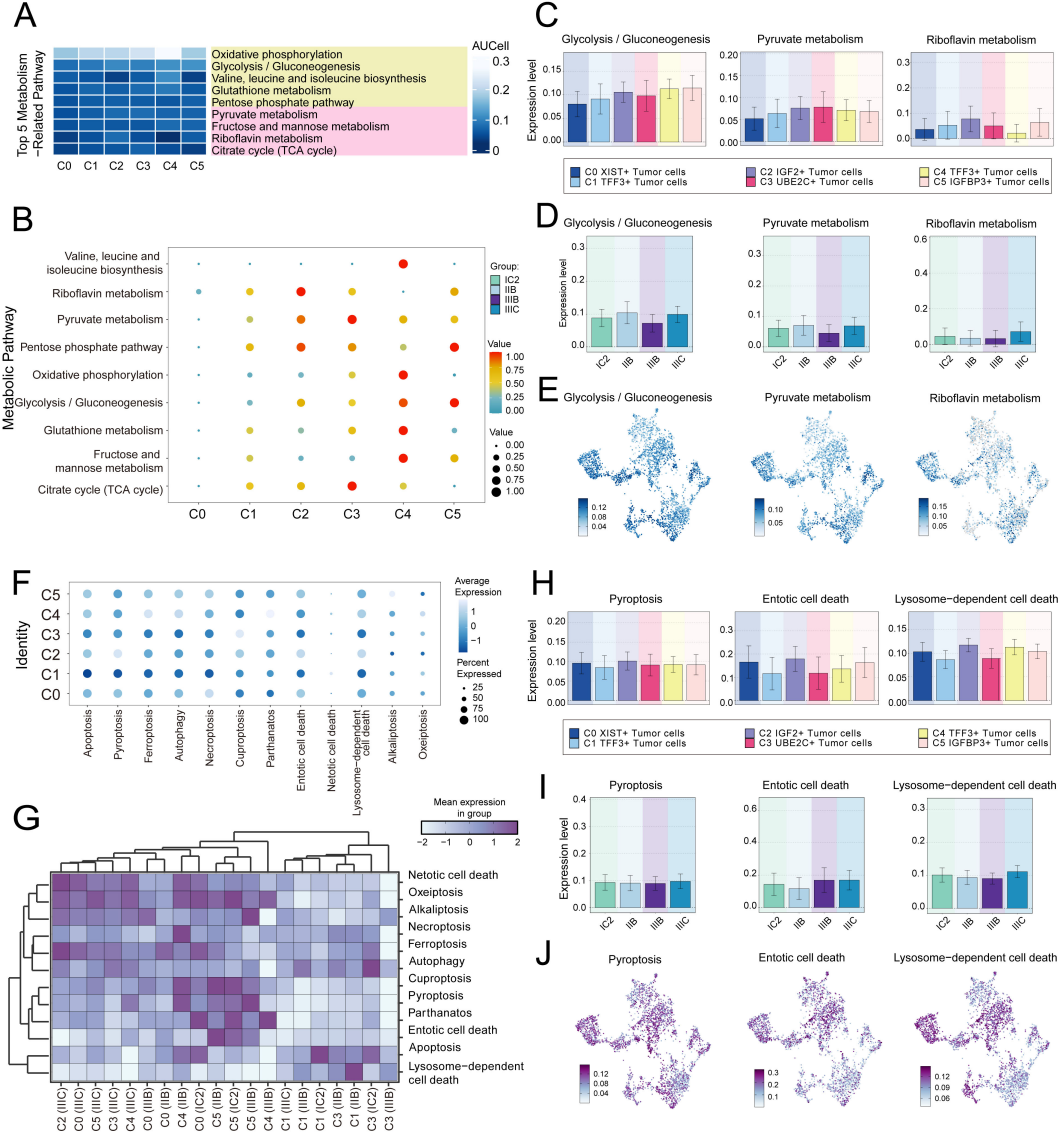
Metabolic and cell death-related gene set analysis in Tumor cells of HGSOC. **(A)** The heatmaps displayed the AUCell scores for the top five metabolism-related pathways across six tumor cell subtypes. **(B)** The bubble plot illustrated the differences in metabolic pathways among the distinct cell subtypes. **(C-E)** The AUCell algorithm was employed to calculate the activity of glycolysis/gluconeogenesis, pyruvate metabolism, and riboflavin metabolism among various cell subtypes and groups. Additionally, UMAP analysis displayed the distribution of these metabolic pathways. **(F, G)** The expression levels of cell death-associated genes in tumor cells from various subtypes were assessed based on the mean gene expression levels. **(H-J)** The AUCell algorithm was employed to calculate the activity of pyroptosis, entotic cell death, and lysosome-dependent cell death among various cell subtypes and groups. Additionally, UMAP analysis displayed the distribution of these cell death-associated genes.

Subsequently, we determined that the subtypes had specific biological functions through the enrichment analysis of tumor cells from different tissue types. For instance, extracellular structure organization, extracellular matrix organization, external encapsulating structure organization was associated with C2 subtype, and the regulation of T cell activation (**Figure 2I, J**). Furthermore, our analysis of the metabolic pathways of different subtypes revealed that the C2 subtype was closely linked to riboflavin metabolism, pyruvate metabolism, and the pentose phosphate pathway (**Figures 2K**, **3A, B**). Further analysis showed that the C2 subtype had higher expression levels in these metabolic processes, and compared to other tissue types, the expression was also more significant in IIIC tissues. The UMAP plot similarly validated our conclusions, clearly presenting the distribution differences of different metabolic pathways across the subtypes (**Figures 3C-E**).

We conducted a comprehensive analysis of biochemical pathways and identified a significant enrichment of the C2 subtype in the programmed cell death pathways of pyroptosis, entotic cell death and lysosome-dependent cell death (**Figures 3F, G**). Our findings indicated that the C2 subtype had higher expression levels in the aforementioned biological processes, and the expression of these processes was also more significant in IIIC tissues compared to other tissue types. The UMAP plot similarly validated our conclusions (**Figures 3H-J**).

### Unveiling the development and differentiation characteristics of tumor cell subtypes through pseudotime analysis

**Figure 4:**
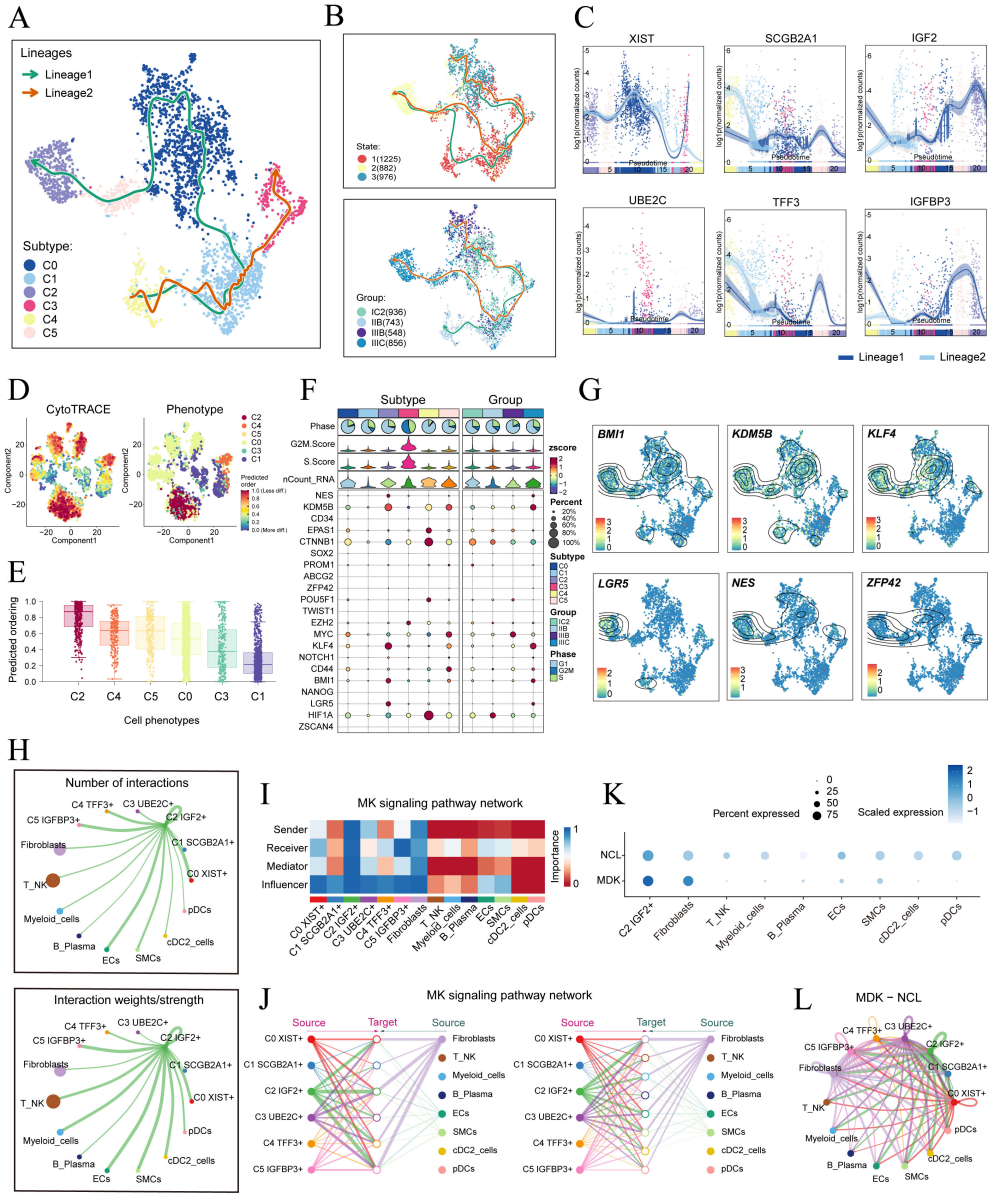
Tumor cell subtypes trajectory analysis and communication crosstalk. **(A)** The UMAP plot showed the differentiation trajectory of six cell subtypes, C2 IGF2+ tumor cells at the end of differentiation. The solid lines represented the differentiation trajectories, and the arrows indicated the direction of differentiation (from naive to mature). **(B)** The UMAP plots showed the differentiation trajectory of across different states and groups. **(C)** The dynamic trend graphs showed the expression of six marker. **(D)** The left panel displayed the genes over time at different differentiation stages.predicted order distribution as estimated by CytoTRACE within tumor cells, where color indicated levels of cell stemness. The right panel illustrated the distribution of tumor cells subtypes, with color representing each tumor cells subtype. **(E)** The Cytotrace analysis was employed to rank the stemness of tumor cell subtypes. **(F, G)** The bubble plot illustrated the differential expression of stemness genes across various tumor cell subtypes and tissue types, and UMAP was employed to visualize the significantly expressed genes. **(H)** Circle plots displayed the number (upper) and strength (lower) of interactions of C2 IGF2+ tumor cells as the source with other cells. **(I)** Heatmap displayed the centrality scores of the MK signaling pathway. **(J)** Hierarchical graph depicted the interactions between C2 IGF2+ tumor cells and other cell types in the MK signaling pathway. **(K)** The bubble plot demonstrated that C2 IGF2+ tumor cells and fibroblasts may interact through the ligand MDK and the receptor NCL. **(L)** The circle plot showed the communication network of MDK-NCL ligand-receptor pairs with tumor cells as the receiver.

To understand the source and development of HGSOC tumor cells, we analyzed the intricate lineage and advancement of tumor cells. It was easy to see in **Figures 4A-D** that the C4 TFF3+ tumor cells were mostly in the early stages of differentiation, Conversely, the C2 IGF2+ tumor cells were in the last stage of development. Afterwards, we utilized the CytoTRACE technique to evaluate the differentiation and developmental correlation between several subtypes of tumor cells. The findings indicated that C2 IGF2+ tumor cells displayed a higher degree of cellular stemness, as seen in **Figure 4E**. Tumor cells often possess self-renewal capability and differentiation potential. Thus, as the tumor advances, tumor cells in the last stage of differentiation tend to have greater cellular stemness, in line with the results obtained from the CytoTRACE investigation. This finding further supported the conclusions drawn from the Monocle analysis.

Consistent with those findings, the genes *IGF2*, which serve as markers for the C2 subtype was predominantly expressed during the mid-late stage of the developmental trajectory. Conversely, the expression levels of *TFF3*, a marker indicative of the C4 subtype, were initially elevated but decreased over time. Subsequently, we illustrated the differential expression of stemness genes across different tumor cell subtypes and tissue types through a bubble plot (**Figure 4F**). Additionally, we visualized the six most prominent stemness genes associated with each subtype (*BMI1, KDM5B, KLF4, LGR5, NES, and ZFP43*) using a combination of UMAP and contour plots (**Figure 4G**).

### Cell-cell communication and visualization of the MK signaling pathway

We utilized CellChat to infer and analyze communication between tumor cell subtypes and other cell types from single-cell data. The number and intensity of interactions between all cell types in HGSOC samples were comprehensively summarized. It was found that compared with other types of cells, C2 IGF2+ tumor cells had a more significant effect on fibroblasts. The circle graphs quantified the number and intensity of interactions between all cells with C2 IGF2+ tumor cells as the signal source and fibroblasts as the target respectively (**Figure 4H**). The results showed that there was a strong intercellular communication network between C2 IGF2+ tumor cells and fibroblasts.

Next, we identified the ligand-receptor signals associated with the communication pathway to determine the primary afferent and efferent signals related to the C2 IGF2+ tumor cells and other cells. Subsequent analysis revealed potential connections to the MK signaling pathway network. By conducting a network centrality analysis of the inferred MK signaling network, we discovered that C2 IGF2+ tumor cells function as signal senders, receivers, mediators, and influencers within the MK pathway. On the other hand, our study found that fibroblasts could function as signal senders, and we hypothesized that this could be related to the transformation of normal fibroblasts into cancer-associated fibroblasts (CAFs). Additionally, fibroblasts could also act as signal receivers, mediators, and influencers, interacting with C2 IGF2+ tumor cells (**Figure 4I**). Notably, C2 IGF2+ tumor cells demonstrated the ability to engage in paracrine interactions with fibroblasts, resulting in a substantial communication intensity between these cell populations (**Figure 4J**). In addition, we compared the receptor-ligand interaction between C2 IGF2+ tumor cells and other cell types and found that when this subtype interacted with fibroblasts, the ligand receptor had a high communication probability with MDK-NCL (**Figure 4K**). Additionally, a circle graph further confirmed that the interactions between C2 IGF2+ tumor cells and fibroblasts could be mediated through the receptor-ligand pairs within the MDK signaling pathway, specifically involving MDK-NCL (**Figure 4L**).

Essentially, our study provided profound insights into the intricate interactions between fibroblasts and tumor cell subtypes in HGSOC. This relationship is likely closely linked to the transformation of fibroblasts into CAFs, which promotes the progression of HGSOC.

### Identification and analysis of TF regulatory modules

TFs can directly interact with the genome and regulate gene transcription by binding to specific nucleotide sequences upstream of the target gene. This interaction plays a significant role in determining the biological functions of cells (62).

**Figure 5:**
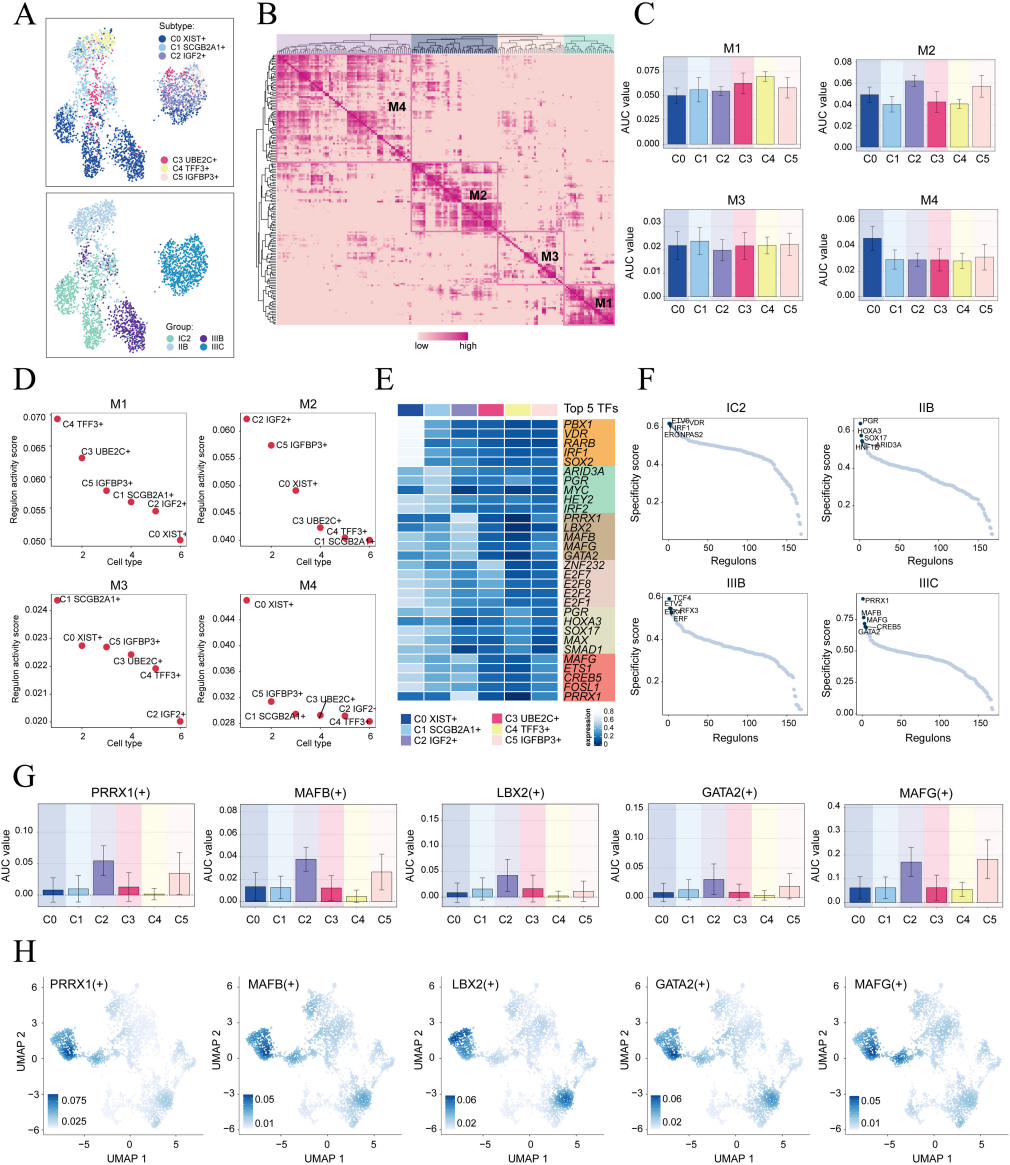
Identification of gene regulatory networks in C2 IGF2+ tumor cells. **(A)** UMAP plots colored and visualized all tumor cells based on the activity scores of regulatory modules, respectively, according to cell subtypes and group classifications. **(B)** Heatmap displayed the identification of four regulatory modules in tumor cell subtypes based on SCENIC regulatory rule modules and AUCell similarity scores. **(C)** The bar graphs showed AUC value of six tumor cell subtypes in four modules comprised by M1, M2, M3, M4. **(D)** The Scatter plots displayed the ranking of TF regulatory activity scores for different tumor cell subtypes in four modules. **(E)** The heatmap displayed top five TFs in six tumor cell subtypes. **(F)** Ranking of the top five TFs activity scores of different group classifications. **(G, H)** Bar plots depicted the AUC value of the top five TFs in C2 IGF2+ tumor cells across different tumor cell subtypes. UMAP plots visualized the distribution of these TFs.

To initiate the analysis, we employed the SCENIC method to re-dimensionally cluster HGSOC tumor cells based on different subtypes and tissue types (**Figure 5A**). Subsequently, we conducted connection specificity index matrix to classify HGSOC tumor cells into four regulatory modules (M1, M2, M3, M4) based on the similarity of AUCell score rules (**Figures 5B**). Through a comparison of the expression levels and regulatory activities of TFs within each module and the tumor cell subtypes, we identified that the TFs in the M2 module predominantly regulated C2 IGF2+ tumor cells (**Figures 5C, D**).

Next, we analyzed the top five TFs in different tumor cell subtypes and different tissue types. We specifically studied their specificity scores in different tissues. It is worth mentioning that PRRX1 showed significant expression in both the C2 subtype and the IIIC tissue. Furthermore, within the IIIC tissue, we observed that PRRX1 exhibited the highest specificity score among the subtypes. This finding suggested a strong and specific regulatory relationship between PRRX1 and its target genes, highlighting its potential as a biomarker or therapeutic target (**Figures 5E, F**). Finally, we visualized the expression levels of five key regulatory factors (PRRX1. MAFB, LBX2, GATA2 and MAFG) in the different subtypes. We observed that the expression of PRRX1 in the C2 subtype was significantly higher compared to other tumor cell subtypes (**Figures 5G, H**). Nevertheless, the specific mechanism by which PRRX1 influences HGSOC remains unclear. Therefore, conducting *in vitro* functional experiments to validate the impact of PRRX1 on HGSOC cells is imperative.

### *In vitro* experimental verification

**Figure 6:**
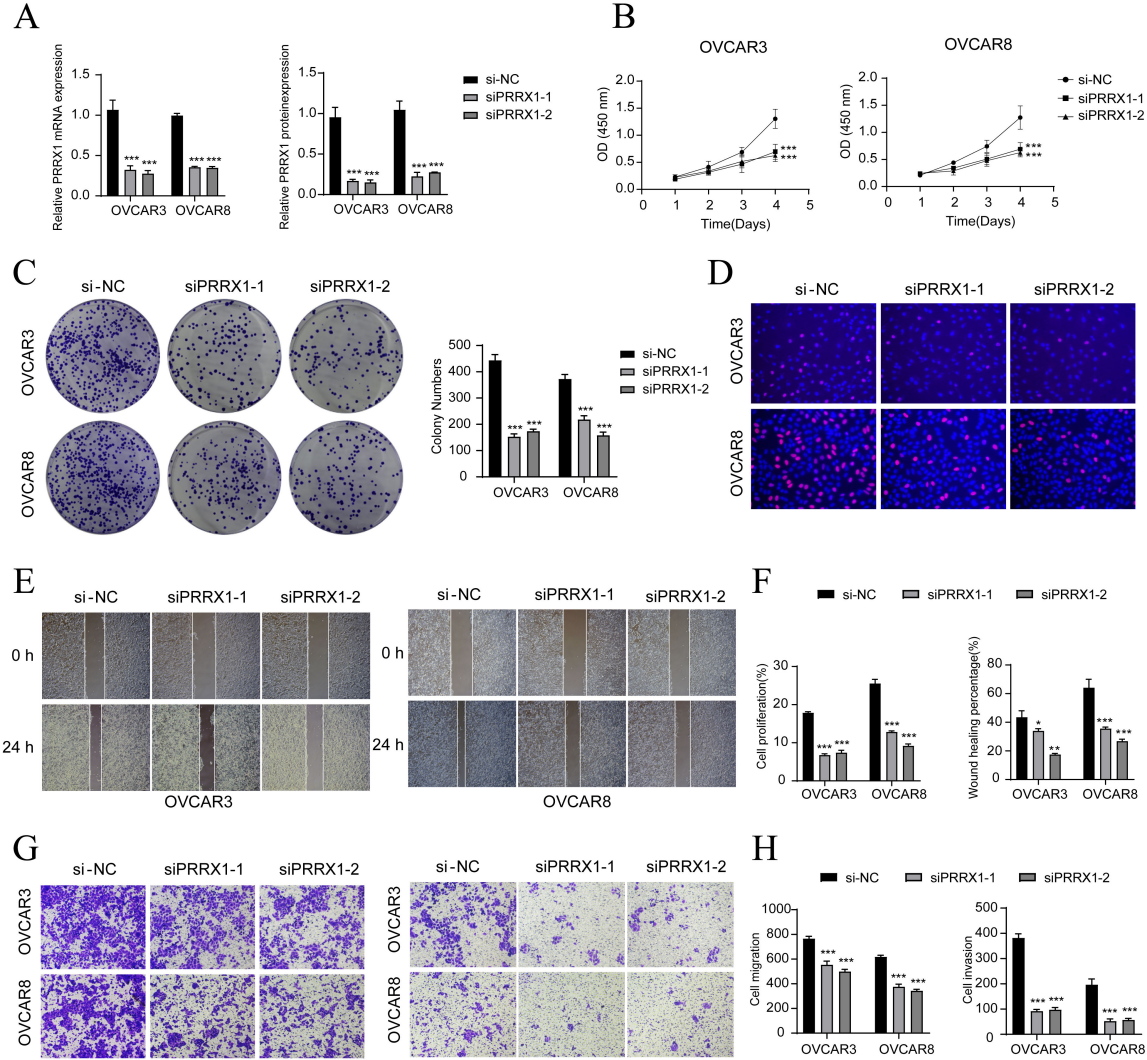
*In vitro* experiments confirmed the effects of PRRX1 knockdown. **(A)** The bar graphs showed the expression of gene mRNA (left) and gene-encoded proteins (right) in the three groups of si-NC, siPRRX1-1, and siPRRX1-2 in OVCAR3 and OVCAR8 cell lines. Following PRRX1 knockdown, both mRNA and protein expression levels were significantly reduced. **(B)** The CCK-8 assay results showed a notable reduction in cell viability in the OVCAR3 and OVCAR8 cell lines following the knockdown of PRRX1. **(C)** Colony formation assays demonstrated a significant decrease in colony numbers after PRRX1 knockdown. The bar graphs showed the colony numbers in two cell lines. **(D)** The EDU staining assay confirmed that PRRX1 knockdown exerted an inhibitory effect on cell proliferation. **(E)** The cell wound healing assays evaluated the migration ability of C2 IGF2+ tumor cells after treatment. **(F)** Bar graph displayed a significant decrease in cell proliferation and wound healing capabilities after PRRX1 knockdown. **(G, H)** Transwell assay showed that PRRX1 knockdown suppressed the migration and invasion abilities of tumor cells in OVCAR3 and OVCAR8 cell lines. *P < 0.05, **P < 0.01, and ***P < 0.001.

To further explore the function of PRRX1 in HGSOC, we performed *in vitro* experiments utilizing OVCAR3 and OVCAR8 cell lines. Initially, we conducted PRRX1 knockdown and evaluated mRNA and protein expression levels both before and after the knockdown. Our results demonstrated a significant reduction in mRNA and protein levels in both cell lines relative to the control group (**Figure 6A**). Additionally, we observed a noticeable decrease in cell viability following the knockdown (**Figure 6B**). Colony formation assays further revealed a substantial decline in cell count after the PRRX1 knockdown (**Figure 6C**). Moreover, EDU and Transwell assays confirmed that the loss of PRRX1 partially impeded cell proliferation (**Figures 6D, G**). The scratch test and Transwell assays also indicated a significant reduction in both cell migration and invasion post-PRRX1 knockdown (**Figures 6E–H**).

To validate our findings, we employed Kaplan-Meier survival curves and ROC curves to analyze the key genes associated with C2 tumor cell subtypes, including the top five transcription factors (IGF2, PRRX1, MAFB, LBX2, GATA2, MAFG). This analysis confirmed the association of IGF2 and PRRX1 with poor prognosis (**Supplementary Figure 2**).

In summary, our results suggest that the knockdown of PRRX1 inhibits the activity, migration, invasion, and proliferation of tumor cells, thereby hindering tumor growth. This inhibition is linked to tumor progression and adverse prognosis.

### Construction and correlation analysis of risk prediction model

**Figure 7:**
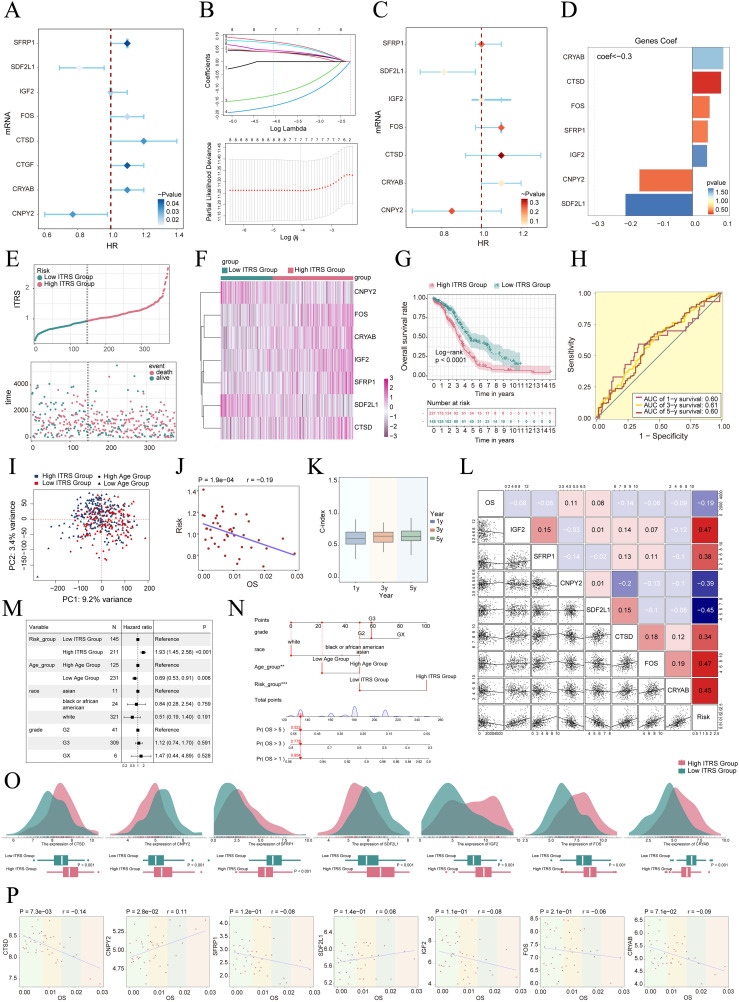
Construction and validation of the IGF2+ tumor cells risk score (ITRS) model. **(A)** Forest plot of univariate Cox regression analysis showing genes with significant differences (HR<1: protective factors, HR>1: risk factors). **(B)** LASSO regression analysis identified eight prognostic-related genes. Each line represents the coefficient of a specific screened to have significant prognostic potential(up). The optimal parameter was determined through cross-validation (upper plot), and the LASSO coefficient curve was determined using the optimal lambda (lower plot). **(C)** Forest plot displayed seven genes obtained from multivariate Cox analysis that were associated with prognosis. **(D)** Bar graph showed the Coef values of the genes used for model construction. **(E)** Curve chart illustrated the risk scores of high and low ITRS groups, and scatter plot depicted survival/death events over time for both groups. **(F)** The heatmap showed the expression of 7 risk genes in the high ITRS group and the low ITRS group, with color scale based on normalized data. **(G)** Kaplan-Meier survival curve illustrated the survival differences among high ITRS group and low ITRS group. **(H)** Calculated the area AUC for predicting outcomes at the 1st, 3rd, and 5th years in the queue. **(I)** Scatter plot showed the distribution of genes along PC1 and PC2 in the high and low ITRS groups. **(J)** The scatter plot showed that risk score was inversely proportional to OS. **(K)** The box plot displayed visualizations of the C-index for cross-validation at 1, 3, and 5 years. **(L)** Heatmap and scatter plots demonstrated the correlation between prognostic genes, OS, and genes used in model establishment. **(M)** The Forest plot demonstrated the results of Multivariate Cox regression analysis integrating risk scores and clinical factors (age, race and tumor clinical stage T, M and N). **(N)** Nomogram showed the prediction of 1, 3, and 5 year of OS based on race, tumor clinical stage (T, M, and N), age, and risk score, with the most significant difference in the risk score group. **P < 0.01, ***P < 0.001. **(O)** Ridge and box plots showed the expression differences of prognosis-related genes in the high ITRS group and low ITRS group. High and low peaks indicate the patient density of patients with this gene expression. **(P)** The scatter plots showed the correlation of seven genes with OS.

We created a prognostic model aimed at investigating the clinical significance of the IGF2+/PRRX1 regulatory network. Initially, we conducted univariate Cox regression analysis to evaluate the individual impact of each gene on prognosis (**Figure 7A**). To address the issue of multicollinearity among the genes, we further employed LASSO regression analysis to select the most relevant genes for prognosis (**Figure 7B**). Subsequently, a multivariate Cox regression analysis was performed, identifying the independent prognostic factors associated with the research outcomes. The results revealed that *CRYAB, CTSD, FOS, SFRP1*, and *IGF2* were identified as unfavorable prognostic factors (HR>1 indicates poor prognosis). Additionally, the coef values for these genes were calculated to quantify their association with survival outcomes (**Figures 7C, D**). Afterwards, using the expression levels and regression coefficients of the five chosen prognostic-related genes, we computed the IGF2+ tumor cells score for each patient using the following formula: IGF2+ Tumor cells score = (0.094017867) × (*CRYAB* expression level) + (0.087558917) × (*CTSD* expression level) + (0.052475918) × (*FOS* expression level) + (0.046824215) × (*SFRP1* expression level) + (0.044591224) × (*IGF2* expression level). These findings emphasize the genes significantly associated with prognosis within the IGF2+/PRRX1 regulatory network.

To delve deeper into the distinctions across various scoring groups, we conducted a DEGs analysis. Utilizing the most favorable cut-off point of the IGF2+ tumor cell score, participants in the TCGA dataset were classified into two distinct groups: those with a high ITRS and those with a low ITRS (ITRS referring to the IGF2+ tumor cells risk score). Our findings indicated that an elevated score correlated with a poorer clinical outcome. Graphs and scatter plots were employed to depict the differences in risk scores, survival rates, and outcomes between the two groups, clearly showing that individuals in the high ITRS group experienced a poorer prognosis (**Figure 7E**). A heatmap was also created to illustrate the differential expression of the seven genes across the high and low ITRS cohorts (**Figure 7F**). The Kaplan-Meier survival curve further validated the finding that the high ITRS group had a significantly worse survival outcome, with a p-value of less than 0.0001 (**Figure 7G**). Additionally, the ROC curve provided a clear visualization of the AUC values predicted by the TCGA cohort at 1, 3, and 5 years, underscoring the model’s predictive capability (**Figure 7H**).

Principal component analysis indicated that PC1, corresponding to the high ITRS group, accounted for 9.2% of the total variance, while PC2, associated with the low ITRS group, explained 3.4% of the variance (**Figure 7I**). We observed that the risk score was negatively correlated with OS, as illustrated in **Figure 7J**, indicating that an increase in risk correlated with a decrease in patient survival time, which aligned with our earlier conclusions. The C-index served as a metric for evaluating the predictive accuracy of the model. Our analysis revealed that, when estimating patient survival at 1, 3, and 5 years, all C-index values exceeded 0.5, indicating a high degree of accuracy in predictions (**Figure 7K**). **Figures 7L-N** presented the assessment of risk, prognostic genes, and hazard ratios based on subgroup analyses, as well as predictions for 1, 3, and 5 year OS across variables such as race, tumor stage (T, M, and N), age, and risk score, highlighting the most significant disparities within the risk score groups.

The results indicate that both the high ITRS group and age are associated with unfavorable prognoses. Further examination of the expression levels of the seven prognostic genes in high and low ITRS groups suggested a more favorable prognosis for *CNPY2* and *SDF2L1*, whereas *CRYAB, CTSD, FOS, SFRP1*, and *IGF2* were associated with poorer prognoses (**Figures 7O, P**).

### Immunoinfiltration, enrichment analysis and drug sensitivity

**Figure 8:**
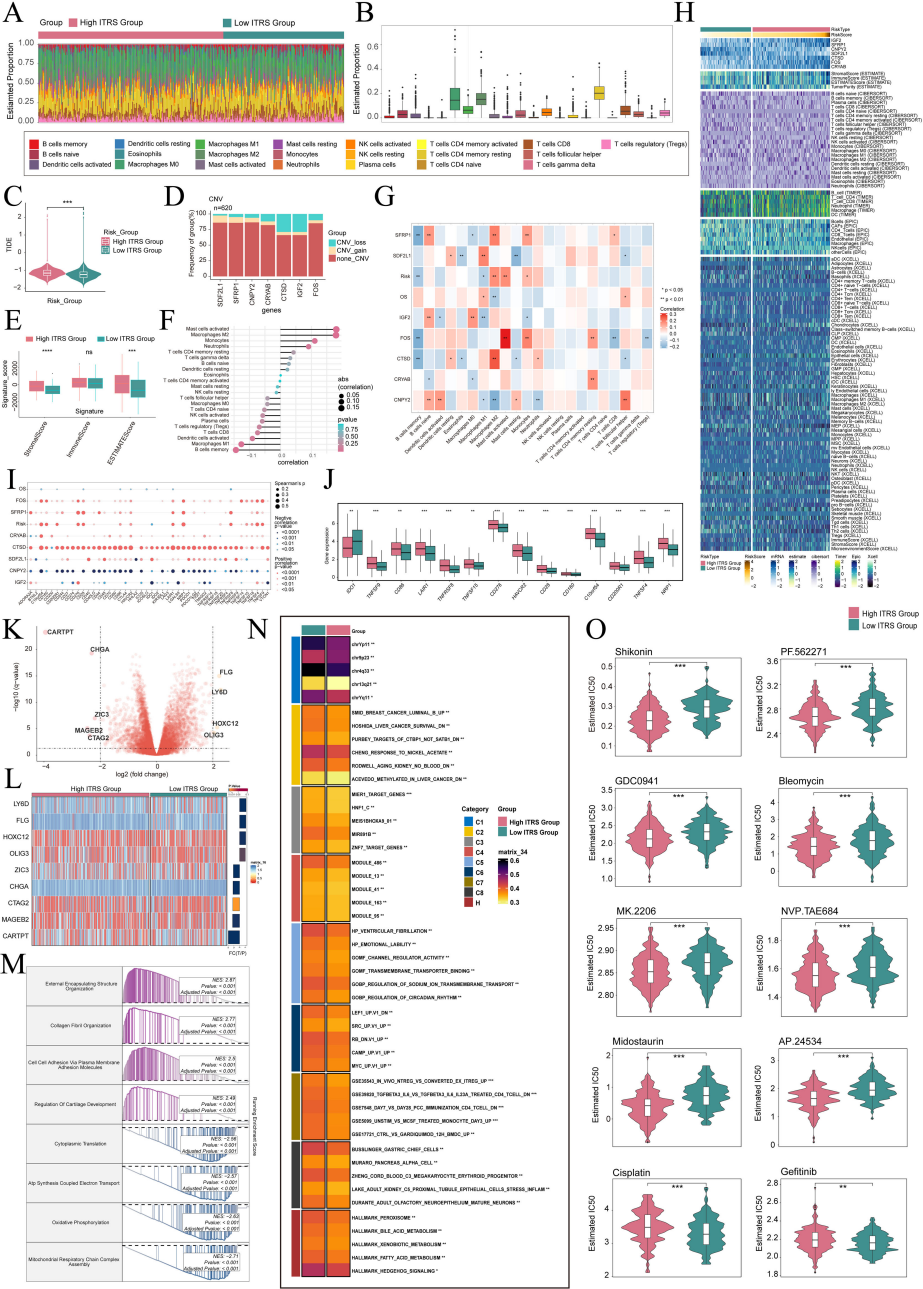
Immunoinfiltration differences, enrichment analysis, and drug sensitivity analysis across different risk groups. **(A, B)** The stacked bar graph and box plot displayed the estimated proportions of 22 types of immune cells among different risk score groups. **(C)** The violin plot illustrated TIDE expression levels in different risk score groups. **(D)** The bar graph depicted CNV gains and losses associated with seven model genes. **(E)** The analysis compared the differences in stromal score, immune score, and ESTIMATE score between the high ITRS group and low ITRS group. **(F, G)** The lollipop chart and heatmap demonstrated the relationship between genes and immune patterns. **(H)** The heatmap highlighted the differences in model gene expression, stromal score, immune score, ESTIMATE score, tumor purity, and levels of immune cell infiltration calculated using CIBERSORT and Xcell between the high and low ITRS groups. Color scales were based on standardized data. **(I)** The bubble plots illustrated correlations among modeled genes, risk scores, OS, and immune checkpoint-related genes. **(J)** The box plot presented the expression levels of immune checkpoint-related genes in both the high ITRS group and low ITRS group. **(K)** The volcano plot showed the significantly upregulated and downregulated genes in the high ITRS group and low ITRS group. **(L)** The heatmap showed the expression of nine DEGs in the high ITRS group versus the low ITRS group. **(M, N)** Detailed results of the GSEA and GSVA enrichment analyses for differential gene sets between the high ITRS group and low ITRS group were presented. **(O)** The violin plots illustrated the differences in IC50 values of various chemotherapy drugs between the high ITRS group and the low ITRS group. *P < 0.05, **P < 0.01, ***P < 0.001, and ****P < 0.0001. "ns" was used to indicate no significant difference.

To elucidate differential gene expression and associated biological processes between high and low groups, we employed visualization and enrichment analysis techniques. Initially, a stacked bar diagram was utilized to show cells estimated proportion in high ITRS group and the low ITRS group (**Figures 8A, B**). There was a slightly higher incidence of TIDE in the high ITRS group (**Figure 8C**). This could have meant that the patients in this group had a higher risk of experiencing adverse events in the near future. A high TIDE score may have indicated the presence of a strong immune-suppressive state in the TME, which could affect the effectiveness of immunotherapy (63). In terms of the level of CNVs of seven prognostic genes, it was observed that *CTSD* and *IGF2* CNV-loss were more frequent (**Figure 8D**). We analyzed the expression levels of the Signature Score in both the high ITRS and low ITRS groups. The results indicated that the high ITRS group had elevated Stromal Scores and ESTIMATE Scores. This finding implied that the high-risk group may possess tumor characteristics associated with enhanced invasiveness or metastatic potential. These aggressive features could have triggered a stronger stromal response, leading to elevated Stromal Scores and ESTIMATE Scores (**Figure 8E**).

Next, we investigated the relationship between these prognostic genes and immune cells and immune processes. It is worth noting that increase in M2 macrophages was associated with the progression of cancer and immune evasion. Notably, a positive correlation between the prognostic model and M2 macrophages, coupled with a negative correlation with M1 macrophages, may provide compelling evidence for immune evasion, disease progression, and unfavorable prognosis in tumors (**Figures 8F-H**). These results enhance our comprehension of the complex interactions among macrophage polarization, the TME, and disease outcomes. Additionally, we conducted an extensive analysis to investigate the relationship between these genes and those associated with immune checkpoints. The findings revealed a positive correlation between *CTSD* and a majority of immune checkpoint-related genes, while *CNPY2* demonstrated a negative correlation with most of these genes. Notably, both *CD276* and *C10orf54* showed elevated expression levels in both the high ITRS and low ITRS groups (**Figures 8I, J**).

Subsequently, we presented volcano plots displaying the upregulation and downregulation of nine DEGs, and utilized a heatmap to illustrate the expression patterns of these genes in the high ITRS group and low ITRS group (**Figure 8K, L**). Afterwards, we applied various enrichment methods to gain further insights into the related biological processes. Specifically, we performed GSEA enrichment analysis on the gene set used in the prediction model, as shown in the enrichment analysis (**Figure 8M**). The GSEA analysis results indicated that the up-regulated genes were significantly enriched in biological processes including external encapsulating structure organization, collagen fibril organization, cell-cell adhesion via plasma membrane adhesion molecules, and regulation of cartilage development. Conversely, the down-regulated genes were mainly enriched in cytoplasmic translation, ATP synthesis coupled electron transport, oxidative phosphorylation, and mitochondrial respiratory chain complex assembly. To achieve a thorough understanding of the functional features and pathway enrichment of tumor cell subtypes with different risk profiles in HGSOC, we performed GSVA enrichment analysis on gene sets corresponding to the high ITRS group and low ITRS group, as shown in **Figure 8N**. Subsequently, we visualized the top-ranked enrichment terms for each gene set and showcased the distribution of risk scores for each enriched term in t-SNE plots (**Supplementary Figure 3A**). The violin plots indicated that the scores for the nine enriched terms were generally higher in the low ITRS group compared to the high ITRS group (**Supplementary Figure 3B**).

Through drug sensitivity analysis, we have identified potential clinical efficacy of certain drugs based on prognosis-related genes. Our study indicates that the high ITRS group exhibits increased sensitivity to chemotherapy drugs including Shikonin, PF562271, GDC0941, Bleomycin, MK.2206, NVP.TAE684, Midostaurin and AP.24534. Additionally, we found that the low-risk group demonstrates lower IC50 values compared to the high-risk group for cisplatin and gefitinib. This finding suggests that cisplatin and gefitinib might lead to better treatment outcomes for low-risk patients, as opposed to the high-risk group, when these drugs are administered (**Figure 8O**).

## Discussion

HGSOC is a highly aggressive subtype of ovarian cancer and belongs to Epithelial Ovarian Cancer. It is characterized by a high degree of clinical heterogeneity, large individual differences, and unsatisfactory therapeutic effects (64). Different tumor cell subtypes may have responded differently to treatment. Notably, we identified a significant association between the C2 IGF2+ tumor cell subtype and stage IIIC tissue, suggesting a pivotal role for this subtype in HGSOC. In stage IIIC of cancer, mainly composed of fibroblasts and ECs, indicating that they might play a key role in the development of late-stage tumors. The activation and interaction of these cell types might promote the invasiveness and therapeutic resistance of tumors through various mechanisms (65).

C2 IGF2+ subtype was a specific type of tumor cells, which exhibited particular gene expression patterns and biological characteristics in tumor cells. Our research has found that this subtype was associated with a higher CNVscore, which could affect the gene expression and function of tumor cells, thereby promoting the invasiveness of the tumor and resistance to treatment. Furthermore, tumors with a high CNVscore were often associated with greater genomic instability, which could lead to more aggressive tumor behavior, such as rapid growth and metastasis. A high CNVscore was typically linked to increased tumor invasiveness and a poorer prognosis (66).

In the C2 IGF2+ subtype, the expression level of IGF2 was typically higher, which might be related to its role in the proliferation and maintenance of stem cell characteristics in tumor cells. IGF2 was a growth factor that activated downstream signaling pathways, such as PI3K/AKT and MAPK/ERK, by binding to the IGF1 receptor and insulin receptor. These pathways played a key role in cell proliferation, survival, and metabolism (67). Moreover, the high expression of IGF2 might also be related to the maintenance of tumor stem cells, which had the ability to self-renew and differentiate into multiple lineages, and were one of the main reasons for tumor recurrence and drug resistance (68).

Other studies have found tumor cells underwent metabolic reprogramming to meet their rapid proliferation needs, a process involving the activation and inhibition of various metabolic pathways (69). For example, glycolysis and oxidative phosphorylation were common metabolic pathways in tumor cells, providing not only energy but also precursor molecules required for biosynthesis. Specific tumor subtypes, such as the C2 subtype, might exhibit unique characteristics in these metabolic pathways, which could be related to their biological behavior and response to treatment (70).

Previous research identified riboflavin, also known as vitamin B2, as a heat-stable, water-soluble vitamin utilized by the body to convert carbohydrates, fats, and proteins into glucose for energy. Beyond enhancing energy levels, this vitamin served as an antioxidant, supporting the proper function of the immune system, as well as promoting healthy skin and hair. In cases of riboflavin deficiency, the digestion of macronutrients such as fats, carbohydrates, and proteins were impaired, hindering the body’s ability to sustain itself (71). Research had indicated that increased intake of riboflavin might lower the risk of ovarian cancer, while vitamin B6 could also contribute to a reduced risk of the disease (72). Additionally, other studies suggested that insufficient folate consumption was linked to a higher risk of developing epithelial cancers, such as colorectal and cervical cancers (73). Further research had shown that higher serum riboflavin levels are linked to a greater risk of pancreatic cancer in a dose-dependent fashion, with a notable effect observed particularly in men ( (74) Riboflavin stimulates the phagocytosis and proliferation of macrophages and neutrophils. In a contrasting effect, it also suppressed the migration and infiltration of neutrophils, as well as the accumulation of activated granulocytes at peripheral locations, potentially leading to a reduction in inflammatory responses (75). Given the close association of riboflavin with immunity, it was reasonable to speculate that the C2 subtype might have exerted a significant influence on immune-related pathways or responses. The presence of riboflavin could have potentially modulated immune cell activity, enhanced immune system function, or affected inflammatory processes. Therefore, the C2 subtype might have had a certain impact on the direction of immune regulation, possibly altering immune cell infiltration, cytokine production, or other key mechanisms involved in immune surveillance and tumor progression. Further research could have clarified the precise role that riboflavin and the C2 subtype played in shaping immune responses.

Similarly programmed cell death, which was recognized as a crucial process in the development and progression of cancer (76). The relationship between the IGF2+ and programmed cell death has been investigated in recent studies (77). Pyroptosis was a form of programmed cell death primarily associated with immune responses. Unlike apoptosis and necrosis, pyroptosis was triggered by the activation of intracellular inflammasomes, which was characterized by cell membrane rupture and a strong inflammatory response (78). In the context of cancer, the role of pyroptosis was multifaceted. Specifically, while pyroptosis could inhibit cancer by eliminating tumor cells and inducing an immune response, it could also, conversely, support tumor growth and metastasis through excessive inflammation (79, 125). Entotic cell death was a form of cell cannibalism, distinguished by one cell engulfing another live cell, which led to its death (80). This phenomenon was first observed in cancer cells, thereby linking it closely to tumor initiation and progression (81). Although it was considered a unique mode of cell death with a critical regulatory role in cancer development, entosis had a dual impact. On the one hand, it could suppress tumors by eliminating abnormal cells (82). On the other hand, it could also promote tumor progression by inducing chromosomal instability and providing cancer cells with a survival advantage (83). Consequently, its role in tumors was complex and varied, depending on the specific cancer type and microenvironment. Lysosome-dependent cell death was a type of regulated cell death triggered by disruptions in intracellular homeostasis and characterized by the rupture of lysosomal membranes (84). Other studies found that lysosome-dependent cell death was a significant mechanism in cancer treatment. Various anticancer agents targeted the lysosomal membrane, causing its disruption and leading to cancer cell death (85). However, some tumors adapted by upregulating lysosomal function, enabling them to resist this form of cell death (86). In our study, we aimed to explore the enrichment of the C2 subtype in programmed cell death pathways, specifically pyroptosis, entotic cell death, and lysosome-dependent cell death. We found that the C2 subtype was significantly associated with these programmed cell death pathways. These pathways are regulated by intrinsic signaling pathways and involve various molecular mechanisms that can be targeted for therapeutic intervention (87). Therefore, the relationship between the C2 subtype and programmed cell death was complex and might have involved multiple factors, including the tumor’s genetic makeup, microenvironment, and the immune response. Further research was needed to fully understand these interactions and to develop effective targeted therapies.

The CytoTRACE technique’s support for these findings was crucial, as it provided a quantitative measure of cellular stemness, a key feature of aggressive cancer cells. The identification of C2 subtype through Slingshot analysis further emphasized the high degree of malignancy in terminally differentiated cells. The ordered developmental trajectory of cancerous cell subtypes from C4 to C2 suggested a progression towards higher malignancy, which was a critical aspect of tumor evolution. The expression patterns of cluster-specific genes like IGF2 and TFF3 were indicative of the cells’ positions within this trajectory, with IGF2 being more prevalent in later stages and TFF3 in earlier stages. In oncology studies, cancer cells at the final stage of differentiation often showed considerable heterogeneity and were associated with greater tumor aggressiveness and difficulties in treatment (88).

We screened for the *BMI1, KLF4*, *LGR5, NES* and *ZFP4*2 stemness gene, Additional research has discovered that the polycomb complex protein *BMI-1* is increased in tumors with proficient homologous recombination. It has also been found that a higher level of *BMI-1* is associated with a worse prognosis in terms of OS for patients with homologous recombination, but this correlation is not observed in patients with homologous recombination deficient HGSOC (64). Exogenous expression of *Klf4* significantly inhibited cell proliferation (89). The expression of nuclear *LGR5* appeared to be protective in terms of OS (90). The structural features of *ZFP42* imply that it might have a transcriptional regulatory function that played an important role in determining the state and developmental stage of stem cells (91). These genes were known to be associated with stemness and malignancy, further supporting the characterization of the C2 subtype as highly malignant. In summary, the comprehensive view of the C2 subtype’s developmental trajectory and malignant potential, as provided by the integration of pseudotime analysis and computational techniques, was essential for understanding the role of this subtype in tumor progression. This understanding not only enhanced our knowledge of cancer biology but also offered potential targets for therapeutic intervention, which could lead to more effective treatments for patients with advanced cancer (92).

In the communication network between C2 IGF2+ tumor cells and fibroblasts, key signaling pathways such as the MK pathway was identified, revealing the complex interactions between tumor cells and stromal cells in the TME. This paracrine signaling exchange may have played a crucial role in the formation of tumor-supporting stroma, affecting the invasiveness, metastatic potential, and response to treatment of the tumor. MDK was a heparin-binding growth factor that interacted with NCL, a multifunctional phosphoprotein present on the cell surface and in the cell nucleus. The MDK-NCL pathway played a key role in the proliferation, migration, and invasion of tumor cells (93). In C2 IGF2+ tumor cells, the expression of MDK might have been upregulated, which could promote the activation and transformation of fibroblasts by binding to NCL on fibroblasts, thus forming CAF that support tumor growth and progression. Specifically, this crosstalk between tumor cells and fibroblasts might have been a key mediator of immune suppression and pro-angiogenic activities in the TME. For example, CAFs could suppress the activity of immune cells by secreting immune-suppressive factors, such as Transforming Growth Factor-beta and Interleukin-10, thereby promoting the immune evasion of the tumor (94). In addition, CAFs could also promote the formation of new blood vessels by secreting angiogenic factors, such as vascular endothelial growth factor, to provide nutrients and oxygen to the tumor (95).

TFs are pivotal in regulating gene expression within cells, as they can either activate or suppress the transcription of specific genes, thereby affecting essential biological processes such as cell proliferation, differentiation, migration, and apoptosis (96). Prior research has suggested that the interactions between TFs and cell death regulators in the TME may influence the vulnerability of tumor cells to programmed cell death. By targeting these signaling pathways, the effectiveness of cancer therapies could be improved by facilitating tumor cell death (97). Consequently, TFs emerged as significant targets for developing targeted therapeutic strategies, particularly in cancer treatment.

As a TF, PRRX1’s poor prognosis made it an attractive target for targeted therapy (98). PRRX1 might promote gastric cancer lymph node metastasis by regulating EMT, which in turn affected patient prognosis (99). Furthermore, PRRX1 was upregulated in various tumors, including colorectal cancer, pancreatic cancer and other cancers, and its expression was closely linked to malignant characteristics such as tumor cell stemness, invasiveness, and angiogenesis (100, 101). The mechanism of action of PRRX1 in HGSOC remained unclear and required further experimental validation. By modulating the regulatory modules involving PRRX1, new drugs could be developed to inhibit its activity, thereby blocking the proliferation and metastasis of tumor cells and improving patient treatment outcomes. We analyzed the TF regulatory network of HGSOC and found that PRRX1exhibited high specificity and expression levels in the C2 subtype, and its correlation with poor prognosis suggested that it may have played a significant role in the development and progression of cancer. Therefore, PRRX1 was not only considered a potential biomarker for predicting patient outcomes and disease progression but also a potential therapeutic target that could be inhibited by targeted therapy to suppress tumor growth and metastasis (102). The results of *in vitro* experiments further supported this viewpoint. Studies showed that after knocking down the expression of PRRX1 using RNA interference technology, the proliferative and migratory abilities of tumor cells were significantly reduced. This indicated that PRRX1 played a key role in regulating the biological behavior of tumor cells, and the loss of its function could lead to a decrease in the proliferative and invasive capabilities of tumor cells.

The development of a prognostic model based on tumor cell subtypes offers a personalized approach to predicting patient outcomes and guiding treatment decisions. The model’s ability to distinguish high-risk patients suggests its utility in stratifying patients for clinical trials and routine care (103). The identification of differential drug sensitivities between risk groups provides a foundation for tailored treatment strategies (104, 105). High-risk groups, for instance, may benefit from specific drugs like Cisplatin, while others might require alternative therapeutic approaches.

Immune checkpoints played a crucial role in modulating immune responses, with tumor cells often evading immune detection by upregulating these checkpoints, which in turn dampened local immune activity (106). Given the high abundance of immune cells in the TME of HGSOC, we investigated variations in immune cell infiltration among different risk assessment groups. The high ITRS group exhibited significantly greater infiltration of naive B cells and resting CD4+ memory T cells compared to the low ITRS group. Naive B cells, a subtype of B cells, have the capacity to differentiate into mature B cells that produce antibodies upon encountering pathogens. In contrast, resting CD4+ memory T cells represent long-lived T cells that develop following prior infections or vaccinations. The presence of these immune cells within the TME, particularly naive B cells and resting CD4+ memory T cells, profoundly influences tumor growth and metastasis, potentially indicating an immune response associated with immune evasion (107). We posited that the increased infiltration of naive B cells and resting CD4+ memory T cells may reflect an immune response related to the immune evasion process (108). Elevated TIDE scores indicated a higher probability of tumor immune escape, suggesting that patients may experience a reduced response to immune checkpoint inhibitor therapies (109). Our findings revealed that the high ITRS group had elevated TIDE scores, further implying a stronger capacity for immune escape within this group.

Macrophages, which were a part of the innate immune system, could change their functions based on the signals they received from their surroundings. There was significant interest in manipulating these cells to reduce inflammation by shifting them from a pro-inflammatory (M1) state to an anti-inflammatory (M2) state, which could have been beneficial for treating inflammatory diseases (110). Subsequently, we evaluated the correlation between immune cells and ITRS. The results demonstrated a significant positive correlation between ITRS and Mast cells activated, Macrophages M2, and a negative correlation with B cells memory, Macrophages M1, among others. In the course of tumor development, M2 macrophages might adversely affect prognosis by supporting tumor growth, angiogenesis, immune evasion, and resistance to therapies. On the other hand, M1 macrophages typically possess anti-tumor properties, inhibiting tumor progression by stimulating inflammation and activating the adaptive immune system. Targeting M2 macrophages could represent a viable treatment approach, while boosting the anti-tumor function of M1 macrophages may further improve patient outcomes. Notably, an inverse correlation was observed between the predictive model score and the levels of M1 and M0 macrophages, implying that tumors might have encouraged the shift of macrophages towards the M1 phenotype Such alterations may have been associated with tumor advancement and unfavorable outcomes.

Additionally, our drug sensitivity analysis revealed differences in the sensitivity of specific drugs between different risk score groups, which may aid in the development of personalized treatment strategies in the future. HGSOC exhibited substantial heterogeneity, with tumors from different patients revealing distinct molecular characteristics and varying sensitivities to drugs. As a result, precision medicine was deemed crucial for the management of HGSOC. Our drug sensitivity analysis indicated that Shikonin, PF562271, GDC0941, Bleomycin, MK.2206, NVP.TAE684, Midostaurin and AP.24534 likely showed enhanced efficacy in patients classified in the high ITRS group.

Shikonin, a natural compound from the Lithospermum plant, had its antitumor activity validated in several studies (111). It was discovered in other studies that shikonin enhanced chemotherapy effectiveness by inhibiting DNA damage response DDR and reducing DNA damage response activation caused by different chemotherapeutic agents in various cancer cell lines (112). Coincidentally, our findings reveal that patients in the high ITRS category exhibit greater sensitivity to Shikonin, aligning with previous research. Similarly, PF562271, a FAK inhibitor, potentially improved drug sensitivity through enhanced immune responses (113). Studies showed that PF562271 effectively suppressed OCa cell growth following chemotherapy (112). GDC0941 exhibited preliminary activity in OCa patients, particularly those with PI3K amplification or PTEN loss (114). GDC0941 was believed to have the potential to deliver better outcomes for OCa patients who had been treated multiple times and showed diminished sensitivity to conventional therapies. By combining these insights, it appeared that both compounds played a role in improving the efficacy of chemotherapy, albeit through different mechanisms: DDR inhibition and immune modulation and direct tumor suppression. This supported further investigation into combination therapies for more effective cancer.

Meanwhile, Bleomycin inhibited cancer cell proliferation by damaging DNA and showed promising efficacy across various cancers (115). MK-2206 specifically targeted the AKT signaling pathway, which could have increased the sensitivity of BRCA-deficient tumors to cisplatin and Olaparib (116). Additionally, NVP-TAE684 (Cabozantinib), another multi-targeted tyrosine kinase inhibitor, demonstrated antitumor activity in other cancers (117). Midostaurin acted as a multi-targeted tyrosine kinase inhibitor, primarily used for certain leukemias (118). Although its application in OCa was limited, the significant role of tyrosine kinase inhibitors in antitumor activity suggested it might serve as a new treatment option for high ITRS patients in the future (119).

Although the specific antitumor mechanisms of AP-24534 remained to be fully elucidated and warranted further investigation (120), our study indicated that this drug exerted significant effects with a relatively low IC50 value in the high ITRS group. This finding highlighted AP-24534’s potential as a novel therapeutic target for advanced HGSOC. Future research should have focused on clarifying the underlying mechanisms of action and evaluating its clinical efficacy in patient populations, thereby facilitating

Although chemotherapy plays a role in the treatment of ovarian cancer, its limitations such as drug resistance, adverse effect, high recurrence rates, and the specific characteristics of HGSOC underscore the necessity for ongoing research into new therapies and medications to enhance treatment efficacy and improve quality of life. Notably, existing studies on chemotherapeutic agents predominantly focus on ovarian cancer as a whole, with insufficient attention given to HGSOC, which is associated with a more aggressive clinical course and poorer prognosis. If the aforementioned drugs have not been studied within the context of advanced HGSOC, it is crucial to recognize this research gap. Future investigations should aim to address this shortfall by exploring tailored therapeutic strategies to augment treatment responses and ultimately improve patient outcomes.

Single-cell sequencing technology transformed biological research by enabling the detailed analysis of individual cells. This advancement provided critical insights into cellular heterogeneity and the complex molecular mechanisms underlying diseases like HGSOC. In the context of personalized treatment, single-cell analysis opened new avenues for understanding the tumor microenvironment and identifying specific cell types or subpopulations that contributed to disease progression or therapeutic resistance. By allowing a more nuanced view of tumor heterogeneity, single-cell analysis enhanced our understanding of HGSOC biology. The identification of the C2 IGF2+ tumor cell subtype, in particular, presented a valuable opportunity for future clinical research. Investigating this subtype led to the development of targeted approaches for early screening and treatment strategies, including the identification of potential biomarkers to enhance detection capabilities and therapeutic targets to improve treatment effectiveness. Additionally, comprehending the unique characteristics of the IGF2+ subtype facilitated patient stratification and enabled personalized treatment decisions, ultimately leading to better patient outcomes. However, this study had several important limitations. Firstly, the sample size was relatively small, focusing primarily on single-cell data from a subtype of HGSOC patients, which may have limited the generalizability of the results. Secondly, the analytical methods relied mainly on single-cell sequencing and transcriptomic analysis without considering other influencing factors. Therefore, future research needed to conduct multicenter studies with larger sample sizes to validate the roles of PRRX1 and the prognostic model in HGSOC. Moreover, incorporating proteomics and metabolomics approaches provided deeper insights into the functional characteristics of specific subgroups, offering a more comprehensive basis for early diagnosis and individualized treatment strategies for HGSOC. Through multi-omics analysis, the biological mechanisms of tumors and potential therapeutic targets were better understood. In summary, our research focused on the diversity of tumor cells in HGSOC at the individual cell level, further revealing the significance of PRRX1. We also identified several prognostically relevant genes, finding a significant correlation between higher ITRS and poorer prognosis. These findings enhanced our understanding of HGSOC development and offered new opportunities for predicting and diagnosing the disease. Future studies should explore these discoveries to advance research and treatment in HGSOC. Collectively, the integration of scRNA-seq into basic and translational research promoted personalized therapy by identifying potential treatment targets for the development of novel drugs and revealing promising biomarkers to monitor treatment efficacy and guide therapeutic decision-making.

## Conclusion

In conclusion, our findings underscored the important role of the C2 IGF2+ tumor cell subtype in HGSOC, particularly its link to advanced disease stages and resistance to therapy. This subtype exhibited distinct gene expression patterns and higher CNVs that contributed to its malignancy and metabolic reprogramming. Additionally, the interactions between tumor cells and the TME, particularly with fibroblasts, suggested potential targets for therapeutic intervention. Future studies should build on these findings to advance HGSOC research and treatment. These insights paved the way for personalized treatment strategies aimed at improving outcomes for HGSOC patients.

## Data Availability

The original contributions presented in the study are included in the article/**Supplementary Material**. Further inquiries can be directed to the corresponding authors.

## Supplementary material

Supplementary Figure 1 The analysis of inferCNV. The analysis of inferCNV. Using scRNA-seq data of ECs to predict CNV. Orange indicated amplification, while blue indicated deletion.

Supplementary Figure 2 The analysis of the C2 subtype marker gene and the top five TFs in bulk. Kaplan-Meier survival curves and ROC curves depicted the marked genes of C2 Tumor cell subtypes and the top five TFs (IGF2, PRRX1, MAFB, LBX2, GATA2, MAFG).

Supplementary Figure 3 Risk scores and differential expression of gene sets. **(A)** The t-SNE plots illustrated the distribution of riskscores, obtained from the top-ranked GSVA enrichment entries for all differential gene sets, within the high ITRS Group and low ITRS Group. **(B)** The violin plots compared the score differences of the aforementioned enrichment entries between the high ITRS Group and low ITRS Group.
