# Supplementary figures and images for: Characterizing tumor biology and immune microenvironment in high-grade serous ovarian cancer via single-cell RNA sequencing: insights for targeted and personalized immunotherapy strategies
Source: Front Immunol. 2025 Jan 17;15:1500153. doi: 10.3389/fimmu.2024.1500153 (PMC11782144; doi:10.3389/fimmu.2024.1500153)

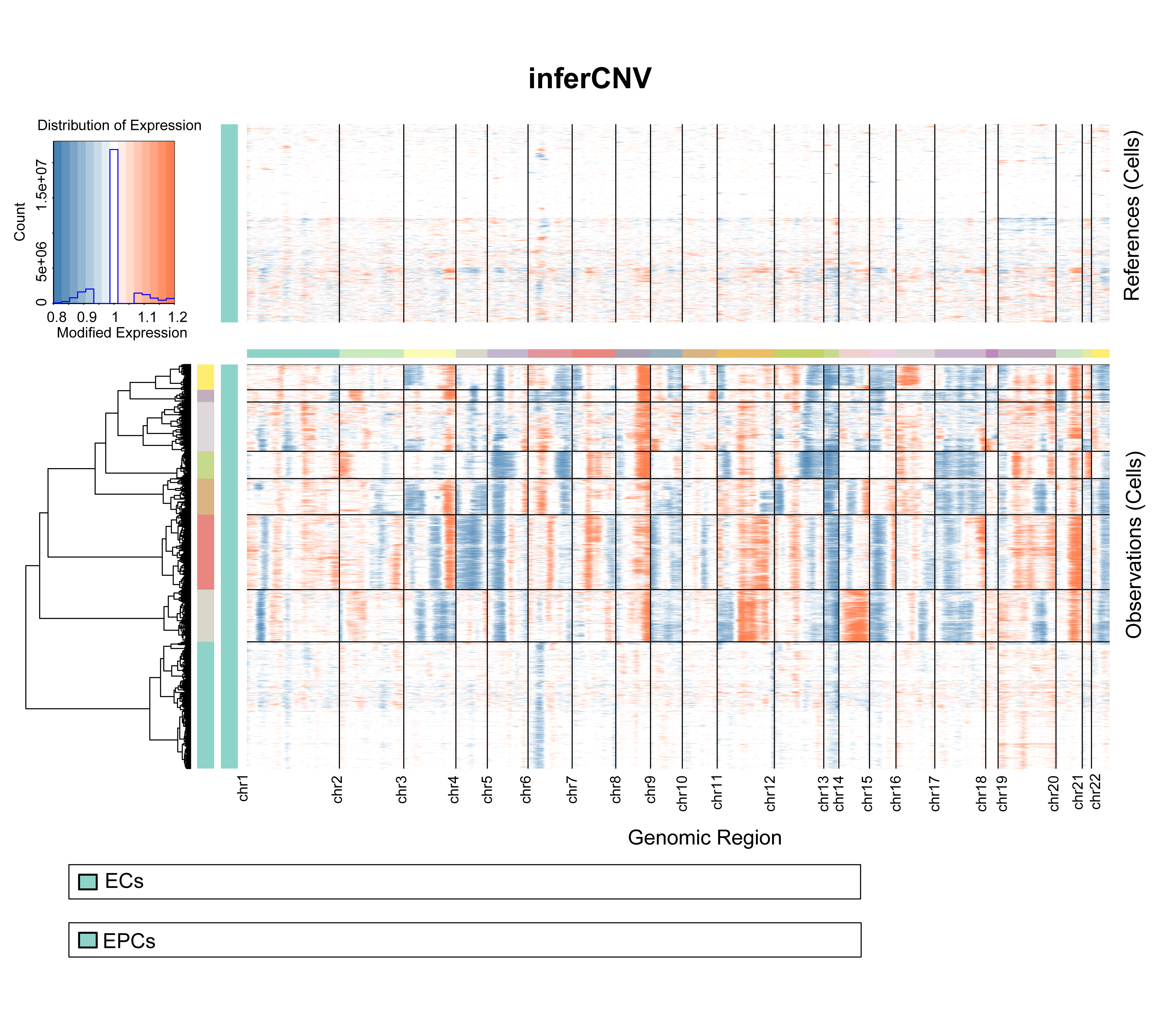

Supplement: Supplementary Figure 1 — The analysis of inferCNV. The analysis of inferCNV. Using scRNA-seq data of ECs to predict CNV. Orange indicated amplification, while blue indicated deletion. [file Image1.jpeg]

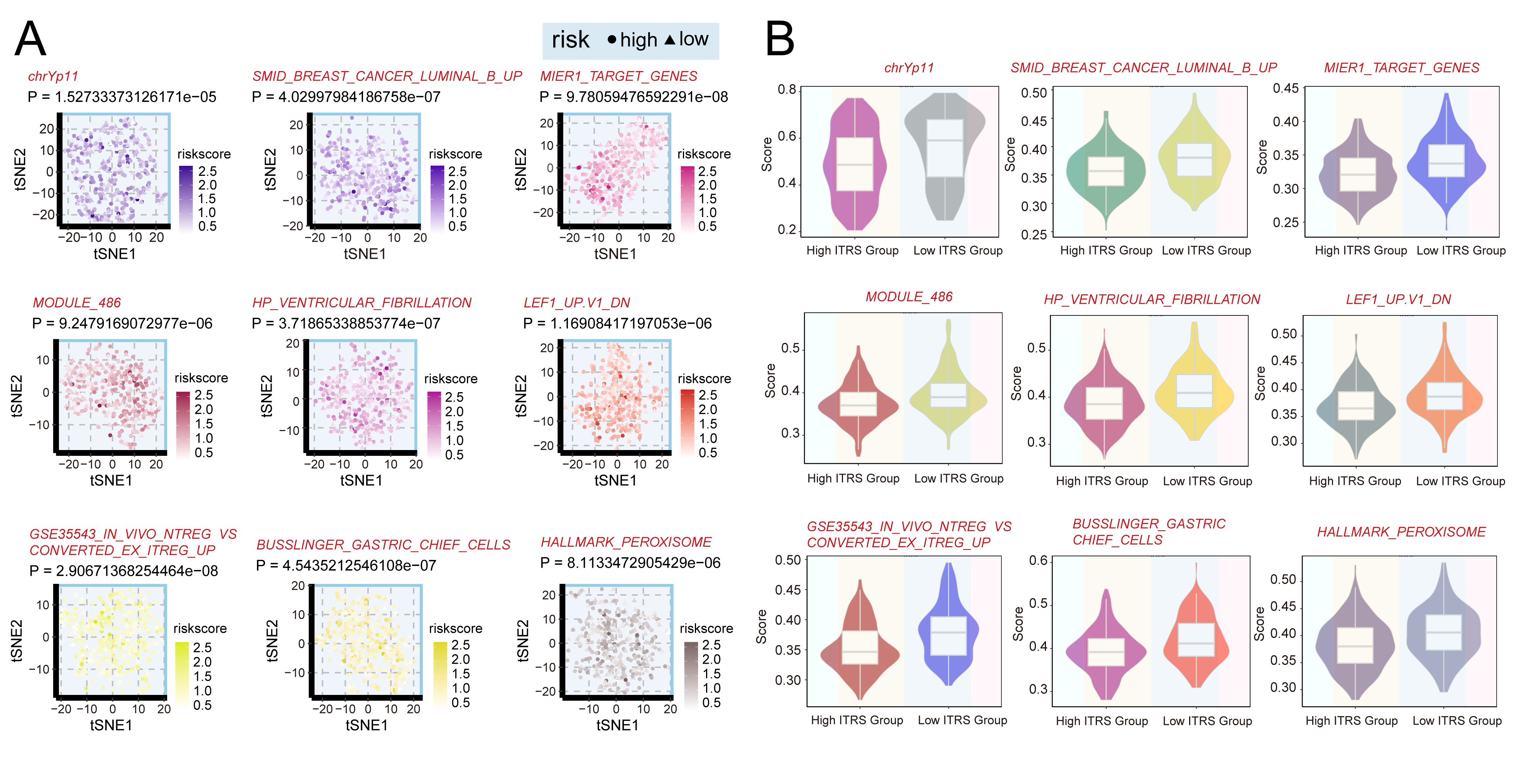

Supplement: Supplementary Figure 2 — The analysis of the C2 subtype marker gene and the top five TFs in bulk. Kaplan-Meier survival curves and ROC curves depicted the marked genes of C2 Tumor cell subtypes and the top five TFs (IGF2, PRRX1, MAFB, LBX2, GATA2, MAFG). [file Image2.jpeg]

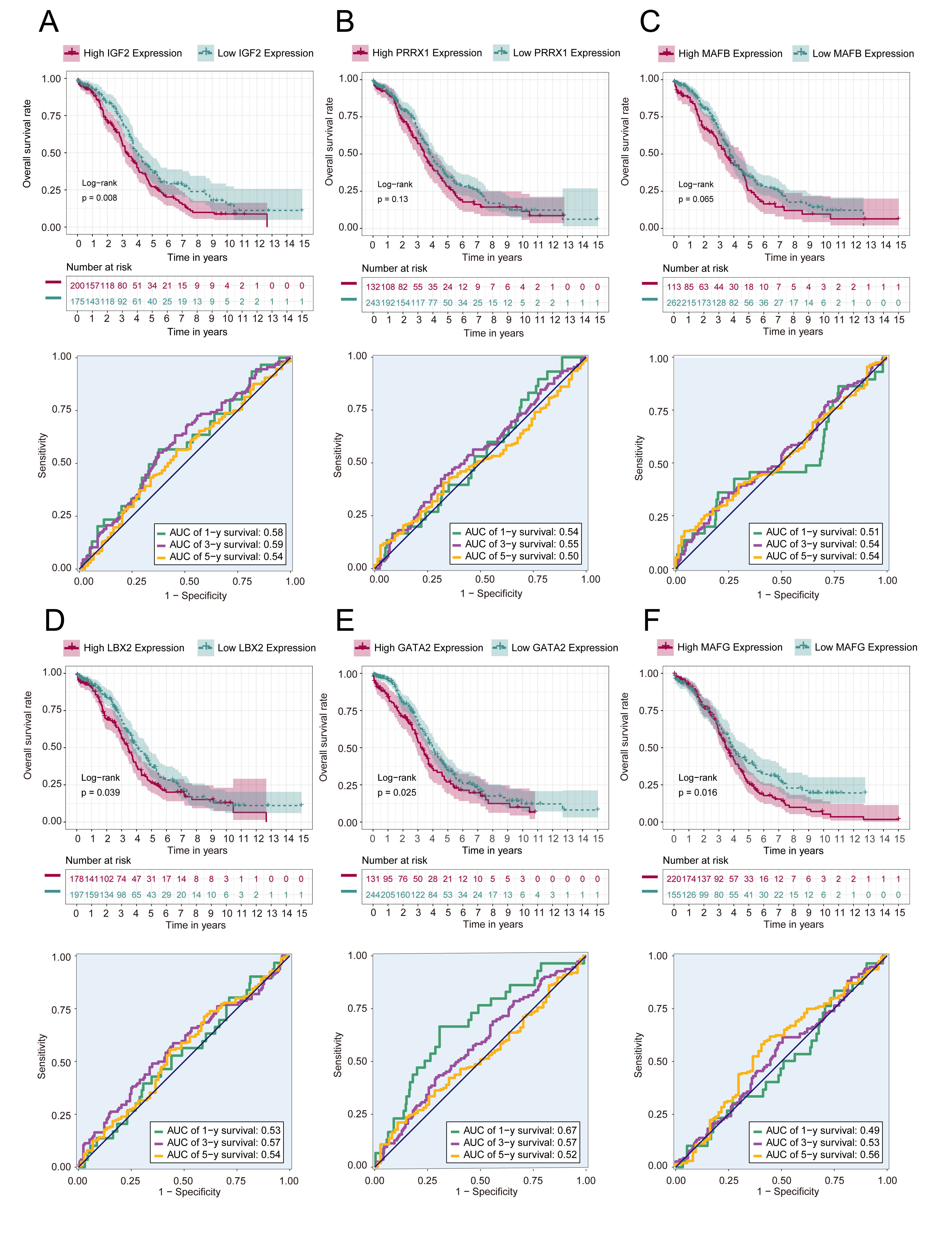

Supplement: Supplementary Figure 3 — Risk scores and differential expression of gene sets. (A) The t-SNE plots illustrated the distribution of riskscores, obtained from the top-ranked GSVA enrichment entries for all differential gene sets, within the high ITRS Group and low ITRS Group. (B) The violin plots compared the score differences of the aforementioned enrichment entries between the high ITRS Group and low ITRS Group. [file Image3.jpeg]
